# Impact of integrase strand transfer inhibitors on cardiovascular disease in people with HIV

**DOI:** 10.1016/j.annepidem.2025.11.006

**Published:** 2025-11-25

**Authors:** Buwei He, Bankole Olatosi, Jiajia Zhang, Sharon Weissman, Xiaoming Li, Xueying Yang

**Affiliations:** a Department of Epidemiology and Biostatistics, Arnold School of Public Health, University of South Carolina, Columbia, SC 29208, USA; b South Carolina SmartState Center for Healthcare Quality, Arnold School of Public Health, University of South Carolina, Columbia, SC 29208, USA; c Department of Health Promotion, Education and Behavior, Arnold School of Public Health, University of South Carolina, Columbia, SC 29208, USA; d Department of Health Services Policy and Management, Arnold School of Public Health, University of South Carolina, Columbia, SC 29208, USA; e Department of Internal Medicine, School of Medicine, University of South Carolina, Columbia, SC 29208, USA

**Keywords:** INSTI, Antiretroviral therapy, Cardiovascular disease, People with HIV

## Abstract

**Purpose:**

Integrase Strand Transfer Inhibitors (INSTIs) are effective and well-tolerated in HIV treatment, but their cardiovascular impact remains uncertain. This study evaluated the association between INSTI-based antiretroviral therapy (ART) and cardiovascular disease (CVD) risk in people with HIV (PWH).

**Methods:**

We conducted a retrospective cohort study using electronic health records and survey data from the All of Us research program. Adults with HIV on continuous ART for ≥ 1 year, free of CVD at baseline and within one year of ART initiation, were included. INSTI exposure was categorized by regimen status. Cox proportional hazards models assessed the association between INSTI use and CVD. A subgroup analysis examined INSTI-naïve individuals who later switched to INSTI-based ART.

**Results:**

Among 2175 PWH, 437 (20.09 %) experienced CVD events; 1715 (78.85 %) used INSTIs. INSTI-only (aHR = 0.65, 95 % CI: 0.50–0.83) and partial-INSTI use (aHR = 0.34, 95 % CI: 0.27–0.42) were linked to lower CVD risk. In the INSTI-naïve cohort (N = 1300), switching to INSTIs reduced CVD risk (aHR = 0.32, 95 % CI: 0.25–0.40). Older age (≥ 70) increased CVD risk.

**Conclusions:**

INSTI-based ART may lower CVD risk among PWH. Further research is needed to validate these findings.

**Summary:**

INSTI-based ART was associated with reduced cardiovascular disease risk among people with HIV, suggesting potential cardioprotective effects that warrant further investigation.

## Introduction

Antiretroviral therapy (ART) is the standard treatment recommended for people with HIV (PWH) [1], with Integrase Strand Transfer Inhibitors (INSTIs) being the preferred option in multiple HIV-related guidelines due to their favorable characteristics [2–4]. Multiple studies have reported that the INSTI-based regimens demonstrate similar or better performance compared to other ART regimens in terms of effectiveness in reducing HIV viral load [5–7], favorable tolerability [5,8–10], and a strong barrier to resistance [11,12]. However, existing research has suggested that INSTIs may be associated with certain complications compared to other ART classes such as Protease Inhibitors (PIs) or Nucleoside Reverse Transcriptase Inhibitors (NRTIs). Metabolic complications, particularly weight gain, have been widely observed [13–15], along with neuropsychiatric effects such as insomnia, anxiety, and dizziness [16,17]. Additionally, gastrointestinal issues, hypersensitivity reactions, and cardiovascular concerns have also been reported [16].

Cardiovascular disease (CVD) remains a leading cause of morbidity and mortality worldwide, with risk factors including hypertension, dyslipidemia, smoking, etc. [18]. PWH face a higher risk of CVD compared to the general population, largely due to a combination of traditional risk factors, chronic immune activation, and the effects of ART [19,20]. As INSTIs become increasingly prevalent in HIV treatment and their associated side effects are reported, assessing the cardiovascular risk of INSTI-based regimens is becoming increasingly important for optimizing long-term treatment strategies and mitigating CVD-related morbidity in PWH.

The cardiovascular safety of INSTI-based ART regimens has been debated, with studies reporting heterogeneous findings. Several large target trial emulations found no significant increase in cardiovascular risk among individuals initiating INSTI-based ART compared with other regimens, regardless of ART experience [21,22]. In contrast, both the RESPOND cohort and the SCOLTA study observed an early excess risk of CVD within the first two years of INSTI exposure [23,24]. Conversely, O’Halloran et al. reported a reduced risk of incident CVD associated with INSTI-based regimens, suggesting possible cardioprotective effects [25]. The heterogeneity in these findings may reflect differences in study design (retrospective vs. prospective cohorts, target trial emulation vs. traditional observational analyses), study populations (ART-naïve vs. treatment-experienced individuals), follow-up duration (short-term vs. long-term exposure), and definitions of exposure or outcomes. Variability in adjustment for baseline comorbidities and cardiovascular risk factors may also have contributed to divergent results. These differences underscore the need for additional analyses that can leverage diverse, real-world data sources and carefully account for potential sources of bias.

Against this backdrop, our study takes advantage of the large All of Us (AoU) Research Program cohort, which includes racially and ethnically diverse populations across the United States [26]. The program deliberately prioritized the enrollment of individuals from backgrounds historically underrepresented in medical research, thereby capturing a broader spectrum of heterogeneity than many traditional cohorts [27]. The breadth of demographic and clinical data, combined with detailed medication histories, further enables evaluation of both initial INSTI use and treatment switches in relation to CVD outcomes within this heterogeneous population.

Building on these insights, our study seeks to further elucidate the relationship between INSTI-based ART regimens and the occurrence of CVD events among people with HIV. Using nationwide EHR data from the AoU research program, we 1) identified individual level ART medication trajectories based on drug exposure records in EHR data; and 2) performed retrospective analyses in all ART-experienced PWH (“general PWH cohort”) and a subgroup of PWH who started ART with a non-INSTI-based regimen (“INSTI-naïve cohort”). Our goal is to complement existing evidence and to provide new insights from a diverse, heterogeneous U.S. population.

## Methods

### Data source

The AoU Research Program is a nationwide initiative launched by the National Institutes of Health (NIH) in 2018 to build a large and diverse biomedical dataset. AoU integrates multiple sources, including electronic health records (EHR), survey responses, physical measurements, biospecimens, and wearable devices, providing a comprehensive platform for studying health outcomes [26]. The AoU cohort is a non-representative sample, characterized by substantial inclusion of groups that have been historically underrepresented in biomedical research. This feature is advantageous for our research question, as it enables the examination of ART trajectories and CVD outcomes in populations that may otherwise be understudied [27]. In this study, EHR and survey data from the Controlled Tier Data (Version 7), encompassing data collected between May 31, 2017, and July 1, 2022, were used to define the study cohort, identify ART medication trajectories, and conduct the analysis.

### Cohort identification

Because consistent HIV identifiers are not available in the AoU data, we applied a computational phenotyping method to define the population of PWH. Participants were classified as PWH if they met at least one of the following criteria: 1) self-reported a prior HIV/AIDS diagnosis in survey responses, 2) had laboratory records indicating a positive HIV test result, 3) had viral load measurements > 200 copies/mL, 4) had HIV-related diagnosis codes, or 5) had prescriptions for HIV-related medications. Details of the phenotyping procedure are described elsewhere [28].

**Fig. 1.**
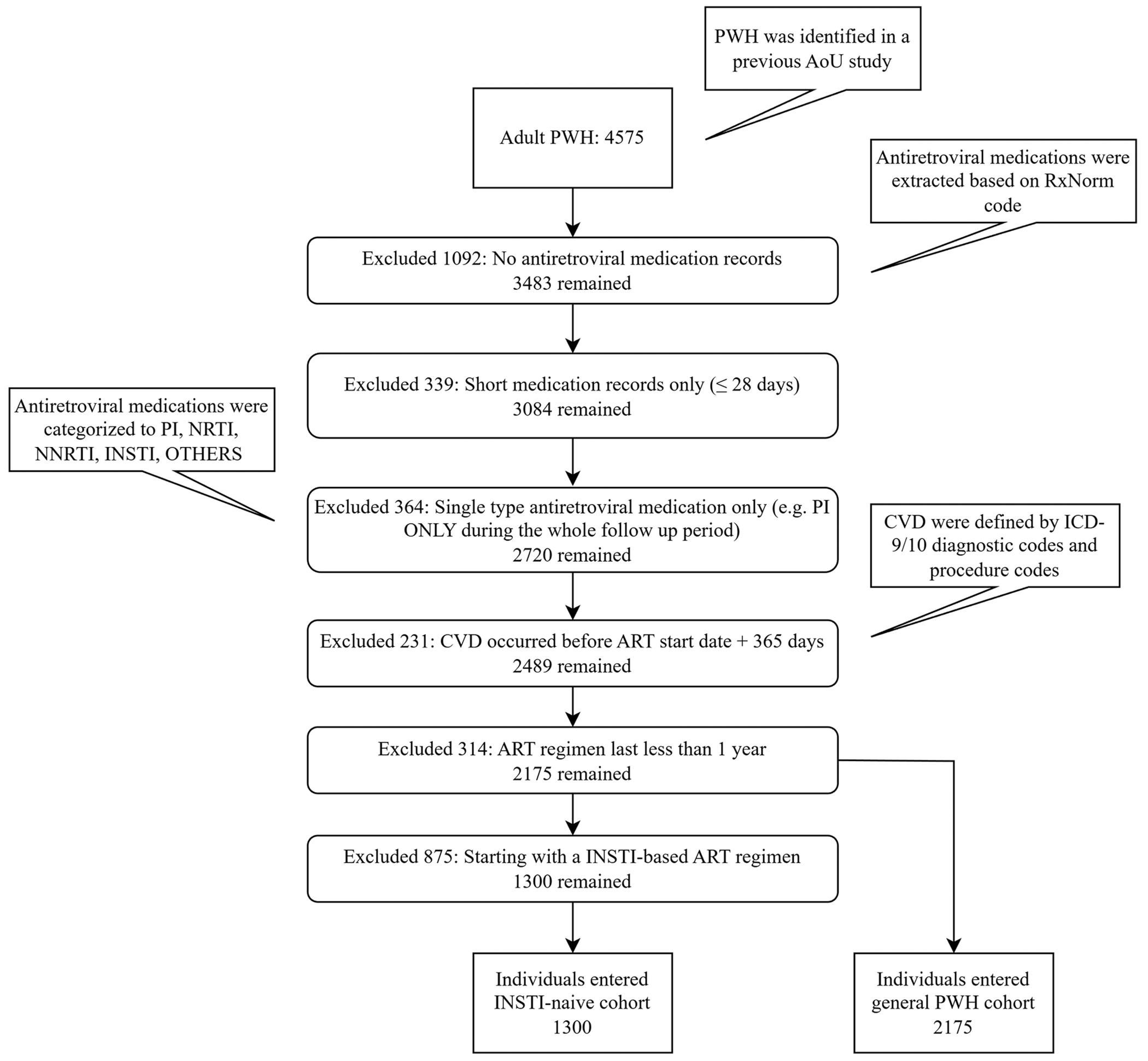
Process of identifying eligible individuals.

In this study, we defined two cohorts to examine the effect of INSTI-based ART on CVD from different perspectives. To examine the overall association between INSTI use and CVD, the general PWH cohort included 1) adult (≥ 18 years old) PWH maintaining eligible ART regimen records for at least a year and 2) participants who were free of CVD before ART initiation and within a year after ART initiation. To specifically assess the effect of switching to an INSTI-based regimen from other ART regimen, the INSTI-naïve sub-cohort applied the same inclusion criteria as the general PWH cohort but was restricted to individuals who initiated ART with a non-INSTI-based regimen. An eligible ART regimen record was defined as a record involving at least two ART drug classes (e.g., NRTI+INSTI, NRTI+INSTI+PI) and lasting for at least 28 days. Accordingly, participants with only short-term ART exposure (< 28 days in all records) or with single-class ART use only (e.g., PI only in all records) were excluded from both cohorts. The detailed cohort construction process is presented in Fig. 1.

### Measures

#### Outcome variable

The outcome was defined as the time to the first CVD occurrence one year after ART initiation. Index time was defined as date of one year after ART initiation, and individuals were followed until the first CVD after the index time, one year after the last ART medication record, or the database end point (July 1, 2022), whichever occurs first. Individuals who were CVD free within the follow-up period were censored at the end of follow-up. CVD was defined as a composite outcome of coronary artery disease or stroke, identified based on the presence of corresponding International Classification of Diseases (ICD)-9 and ICD-10 diagnostic codes and procedure codes (supplementary Table 1).

### Main predictor of interest

The main predictors for both cohorts were defined from the INSTI-based ART usage. Specifically, antiretroviral medications were first extracted from the AoU drug exposure data based on their associated RxNorm codes (supplementary Table 2) [29,30]. These medications were then categorized into five drug classes based on their mechanism of action: PIs, NRTIs, Non-Nucleoside Reverse Transcriptase Inhibitors (NNRTIs), INSTIs, and others. The classification criteria were detailed in the supplementary material (supplementary Table 3). The observation period of the ART drug exposure was from the ART initiation to the end of follow-up for each individual. For the general PWH cohort, the main predictor was the ART regimen type categorized by the INSTI-containing status, i.e., only-INSTI (i.e., only INSTI-based ART regimens were used during the observation period), non-INSTI (i.e., only non-INSTI-based ART regimens during the observation period), and partial-INSTI (i.e., both INSTI-based and non-INSTI-based ART regimens were used during the observation period). For the INSTI-naïve cohort whose ART did not contain INSTI at baseline, the main exposure was switching to INSTI, defined as a change from a non-INSTI-based regimen to an INSTI-based regimen. Detailed data examples for all categories of the predictors were provided in the supplementary material (supplementary Table 4) for better understanding.

### Other covariates

Sociodemographic variables were included as covariates and identified from the survey data in AoU database. They are age at AoU enrollment (e.g., 18–29, 30–39, 40–49, 50–59, 60–69, and ≥ 70 years old), sex (e.g., Male, Female, and Other), race (e.g., White, Black, and Asian/Other/Unknown), ethnicity (e.g., Hispanic, non-Hispanic, and Unknown). Several laboratory test results extracted from the AoU lab and measurements data were incorporated [29], including baseline HIV viral load (VL) (e.g., < 200, 200–10,000, ≥ 10,000 copies/mL, and Unknown), baseline CD4 cell count (e.g., < 200, 200–350, ≥ 350 cells/μL, and Unknown), and baseline body mass index (BMI) (e.g., < 25, 25–30, ≥ 30 kg/m^2^, and Unknown), each defined using the most recent corresponding laboratory record prior to the index time for each individual. Lipid-lowering drug use was defined as a baseline binary variable indicating whether statins were used during the observation period (i.e., from the ART initiation to the end of follow-up). The concept IDs used to identify the lipid-lowering drug from the AoU drug exposure data were listed in the supplementary material (supplementary Table 5). We also adjusted for the number of historical comorbidities (e.g. 0, 1, and ≥ 2) in the analysis. The comorbidities considered included chronic obstructive pulmonary disease, cancer, dementia, diabetes, hemiplegia/paraplegia, liver disease, peptic ulcer disease, renal disease, and rheumatic disease. Each historical chronic condition was defined by the presence of the corresponding ICD codes (supplementary Table 6) prior to the index time for each individual.

### Statistical analysis

Descriptive statistics were first computed to summarize baseline characteristics of the study population. For continuous variables, we reported the sample mean and standard deviation (SD), while categorical variables were summarized using counts and percentages. Differences between individuals who experienced a CVD event and those who were censored were evaluated using Pearson’s Chi-square test for categorical variables and Welch’s two-sample *t*-test for continuous variables. To assess the association between INSTI-based ART regimen and the risk of CVD, we applied Cox proportional hazards (PH) models. Both crude (univariable) and adjusted (multivariable) Cox models were fitted to evaluate the effect of INSTI exposure, adjusting for demographic and clinical covariates. The PH assumption was validated using log-log survival curve graphical method (supplementary Figures 1a and 1b). All analyses were conducted using R version 4.4.0 within the AoU researcher workbench, with statistical significance set at a two-sided p-value of 0.05.

### Sensitivity analysis

Due to the complexity of individual-level drug exposure trajectories, the partial-INSTI category in the general PWH cohort included multiple types of INSTI exposure patterns: 1) switch to INSTI (initiated on a non-INSTI-based ART regimen and later switched to an INSTI-based regimen), 2) switch out of INSTI (initiated on an INSTI-based regimen and later switched to a non-INSTI-based regimen), and 3) multiple switches (a combination of the first two patterns occurring repeatedly during follow-up). Furthermore, baseline characteristics differed significantly across drug exposure categories. To minimize potential bias arising from heterogeneous exposure histories and baseline imbalances, we conducted the following sensitivity analyses to assess the robustness of our findings. First, we excluded individuals who switched out of INSTI or had multiple switches, restricting the partial-INSTI group to those with a single switch to INSTI (hereafter referred to as the switch-INSTI group). Second, we defined the total duration of INSTI-based ART use (in years) as a continuous exposure variable in the general PWH cohort and fitted the model using this continuous measure as the main predictor. Third, to mitigate baseline confounding, we applied propensity score matching (PSM) using a nearest neighbor algorithm with a caliper width of 0.02. Specifically, we conducted 1:1:1 PSM based on multinomial logistic regression in the general PWH cohort and 1:1 PSM based on logistic regression in the INSTI-naïve cohort [31,32]. Baseline variables demonstrating marginally significant differences across drug exposure groups (i.e., age, number of historical comorbidities, lipid-lowering drug use, and baseline viral load/CD4 count/BMI) were included in the construction of the propensity score model.

## Results

### Characteristics of study population

Among 4575 confirmed adult PWH [28], 2175 eligible individuals were included in the general PWH cohort, of whom 460 (21.15 %) experienced a CVD event. Within this cohort, 437 (20.09 %) individuals never used an INSTI-based regimen throughout the observation period, while 783 (36.00 %) used INSTI-based regimens exclusively. The mean age at enrollment was 55 years (SD = 11.74), and the majority of participants were aged 40 years or older (87.77 %). Of the 2175 individuals, 628 (27.87 %) were assigned female at birth, 1165 (53.56 %) were Black, 373 (17.15 %) were Hispanic or Latino, and more than half (59.68 %) had no history of chronic disease. Additionally, 862 (39.63 %) individuals used lipid-lowering medications during the observation period. Among the 1300 individuals in the INSTI-naïve sub-cohort who initiated ART with a non-INSTI-based regimen, 863 (66.38 %) later switched to an INSTI-based regimen during follow-up. Overall, 320 (24.62 %) participants in this cohort experienced a CVD event. The demographic characteristics of the INSTI-naïve sub-cohort closely resembled those of the general PWH cohort. Approximately 90 % were aged 40 years or older at enrollment, 30.92 % were assigned female at birth, and 55.46 % were Black. A higher proportion of non-INSTI-based ART users experienced CVD events in both cohorts (general PWH cohort: 30.66 % non-INSTI vs. 15.45 % only-INSTI and 21.47 % partial-INSTI. INSTI-naïve cohort: 30.66 % non-INSTI vs. 21.55 % switch to INSTI. p < 0.001). Comprehensive demographic and laboratory characteristics are presented in Tables 1a and 1b.

### Factors that impact occurrence of CVD

Factors potentially associated with CVD are summarized in Tables 2a and 2b and Figs. 2a and 2b. In the general PWH cohort, individuals who only used INSTI-based regimens had a longer survival time before experiencing a CVD event compared to those who only used non-INSTI-based regimens (adjusted hazard ratio (aHR) = 0.65, 95 % CI: 0.50–0.83). Individuals who took both INSTI-based and non-INSTI-based regimens (partial-INSTI) exhibited an even stronger protective effect against CVD (aHR = 0.34, 95 % CI: 0.27–0.42). Significant risk effects were observed in the crude model for baseline BMI ≥ 30 kg/m^2^ (hazard ratio (HR) = 1.59, 95 % CI: 1.18–2.15) and in the adjusted model for baseline viral load ≥ 10000 copies/mL (aHR = 1.53, 95 % CI: 1.07–2.20). For the INSTI-naïve cohort, switching to INSTI-based regimen was associated with a lower hazard of experiencing CVD event (aHR = 0.32, 95 % CI: 0.25–0.40). Regarding other covariates, the survival model produced similar results in both cohorts. An increasing hazard with age was observed, indicating that older participants were more likely to develop CVD, although statistical significance was not consistently strong across all age groups. Participants who used lipid-lowering medications during the observation showed a higher risk of developing CVD. Significant effects were also detected for number of comorbidities, with a higher number of comorbidities associated with an increased hazard of CVD. No statistically significant associations were found between sex, race, or ethnicity and CVD outcomes, except in the INSTI-naïve cohort, where individuals who identified as Hispanic or Latino appeared to have a lower probability of experiencing CVD compared to non-Hispanic or Latino individuals (aHR = 0.46, 95 % CI: 0.24–0.88).

The results of the sensitivity analyses were generally consistent with the main findings. After excluding confounding cases from the partial-INSTI group in the general PWH cohort, the protective effect of using INSTI-based ART regimen against CVD remained significant (switch-INSTI: aHR = 0.33, 95 % CI: 0.26–0.42, only-INSTI: aHR = 0.65, 95 % CI: 0.50–0.83). Moreover, a longer cumulative duration of INSTI-based ART use was associated with a lower risk of CVD (aHR = 0.80, 95 % CI: 0.77–0.83). After accounting for baseline differences among drug exposure groups through PSM, the cardioprotective effect of INSTI-based ART regimens remained robust. Detailed results from the sensitivity analyses are presented in the supplementary material (supplementary Tables 7–14).

## Discussion

To the best of our knowledge, this study is among the first to investigate the association between INSTI-based ART regimen and CVD among PWH using EHR data from AoU. We defined two separate cohorts to examine the effect of INSTI-based regimen on CVD occurrence from different perspectives. The general PWH cohort was used to assess the overall effect of INSTI exposure, revealing that ART regimens involving INSTIs had a protective effect against CVD compared to non-INSTI-based regimens. By restricting the analysis to individuals who initially received non-INSTI-based ART, the INSTI-naïve cohort allowed us to explore the impact of switching to INSTI-based regimens, with findings consistently indicating a protective effect on CVD occurrence. These findings persisted after adjustment for demographic and clinical covariates, including baseline BMI, viral load, CD4 count, and lipid-lowering drug use. Not surprisingly, CVD risk increased with older age and greater comorbidity burden. No significant differences in CVD risk were observed across sex, race, or ethnicity groups.

Our findings align with several recent studies examining the cardiovascular implications of INSTIs while adding nuance to the ongoing debate. O’Halloran et al. similarly reported that initiating an INSTI-based regimens was associated with a significantly lower risk of incident CVD compared to non-INSTI-based regimens in a large US cohort (HR = 0.79, 95 % CI: 0.64–0.96) [25]. The cardioprotective effect of INSTI-based regimens may be attributed in part to the favorable metabolic profile of INSTIs: unlike some older antiretroviral medications, INSTIs generally do not worsen serum lipids and often improve lipid profiles when substituted for PIs [33–35]. This could plausibly translate into fewer cardiovascular events over time. Consistent with this notion, switching from PI-based regimens to INSTI-based regimens (e.g. Raltegravir) has been shown to improve dyslipidemia without loss of viro-logic control [36]. Such metabolic advantages may help explain the lower rates of myocardial infarction and stroke observed in INSTI-treated patients in our study and others.

In contrast, several studies have reported divergent findings. For example, Rein et al. and Surial et al. observed no significant difference in CVD risk between individuals receiving INSTI-based ART and those on other regimens [21,22]. Moreover, other studies have reported a higher CVD risk associated with INSTI use [23,24,37]. Several factors likely explain these apparent discrepancies. First, differences in study design and analytic framework play a key role. Neesgaard et al. reported an elevated CVD risk during the first one to two years of INSTI exposure. Specifically, the incidence of CVD among INSTI users was roughly doubled in the first 6 months (adjusted incidence rate ratio = 1.85, 95 % CI: 1.44–2.39) and remained modestly higher through 24 months, before declining to baseline levels by year 3 of INSTI-based therapy. The discrepancy between their results and ours may stem from the differences in study design. Whereas Neesgaard et al. counted CVD events from the moment of ART initiation, our study began observing CVD events one year after ART start, intentionally excluding the first year when biological changes and acute ART effects might contribute to CVD risk [23]. By design, our analysis focused on longer-term outcomes after patients had been on stable therapy for at least one year, which likely mitigated short-term confounding. Second, differences in study populations are also critical. Whereas the Swiss, SCOLTA, and RESPOND cohort included predominantly White European adults, the All of Us dataset encompasses a racially and socioeconomically diverse U.S. population [21,23,24,26]. This broader representation enhances generalizability and captures variations in healthcare access, comorbidity burden, and treatment practice that are underrepresented elsewhere. Third, variability in exposure definitions and modeling approaches could further explain inconsistencies. For example, Surial et al. defined INSTI exposure as receiving any INSTI-containing ART regardless of whether other ART components were co-administered and applied pooled logistic regression for estimation. Although there is no universally accepted definition of a “valid” ART regimen, clinical practice typically involves combinations of drugs from multiple classes. Neglecting this definition may introduce bias by including incomplete or invalid treatment histories. Thus, our setting aligns more closely with clinical guidelines and likely reduced misclassification. Further methodological comparisons and validation studies are warranted to assess model performance in diverse populations and study settings.

This study is subject to several limitations. First, the analysis in general PWH cohort yielded the somewhat counterintuitive result that the “partial-INSTI” group showed a stronger protective effect than the “only-INSTI” group. This pattern was corroborated by our sensitivity analysis, which indicated that switching from a non-INSTI to an INSTI-based regimen conferred a greater reduction in CVD risk than continuous INSTI use compared to non-INSTI users and thus driving the apparent protective effect observed in the partial-INSTI group. While the precise mechanism remains unclear, a plausible explanation involves adverse metabolic profiles associated with prior non-INSTI therapy [38, 39]. Transitioning away from these agents may improve serum lipid levels and overall metabolic health, yielding an apparent cardiovascular benefit after switching. Second, individuals using lipid-lowering medications exhibited higher CVD risk. This counterintuitive association likely reflects confounding by indication, as lipid-lowering drugs are typically prescribed to individuals with elevated cardiovascular risk or pre-existing dyslipidemia. Consequently, medication use serves as a marker of higher baseline risk rather than an independent protective factor. Third, although baseline BMI, viral load, and CD4 cell count were included as covariates, a substantial proportion of participants lacked these measurements and were therefore categorized into an “Unknown” group. While this approach preserved sample size, it may not fully eliminate bias introduced by missing data. Another limitation involves the external validity of our findings. Participants in the AoU program are volunteers who are generally more engaged with healthcare and may not fully represent the broader U.S. population. In addition, our cohort definitions required continuous ART use and at least one year of follow-up, which excluded individuals with fragmented or irregular medical care. These factors improve data completeness but may reduce external validity, meaning our results are most applicable to people with consistent HIV care. Finally, the reliance on EHR data may lead to misclassification of underreporting of CVD and other historical chronic conditions due to the use of ICD codes. Similarly, medication exposure was inferred from prescription records; although we required continuous ART for inclusion, we could not perfectly verify adherence or account for short gaps in drug supply.

## Conclusions

In conclusion, this study provides empirical evidence from a large nationwide cohort of PWH that INSTI-based ART regimens are associated with a lower risk of CVD. Using EHR data from the AoU research program, we identified the cardioprotective effect of INSTI-based regimens on CVD occurrence in two separate cohorts. Switching to an INSTI-based regimen from a non-INSTI-based regimen significantly reduced CVD hazard in INSTI-naïve individuals, while regimens involving INSTIs showed consistent protective effect compared to non-INSTI-based regimens in the general PWH cohort. These findings align with prior research highlighting INSTIs’ favorable metabolic profile, though inconsistencies with studies reporting early CVD risk warrant further investigation. However, given the inherent limitations of EHR data, including potential misclassification and unmeasured confounders, further prospective studies are needed to validate these findings and explore the biological mechanisms underlying the observed protective effects. Future research should also investigate long-term effects of INSTI-based regimens on CVD risk to inform optimal ART management and improve the long-term health outcomes of PWH.

## Supplementary Material

1

2

Supplementary data associated with this article can be found in the online version at doi:10.1016/j.annepidem.2025.11.006.

## Figures and Tables

**Fig. 2a.**
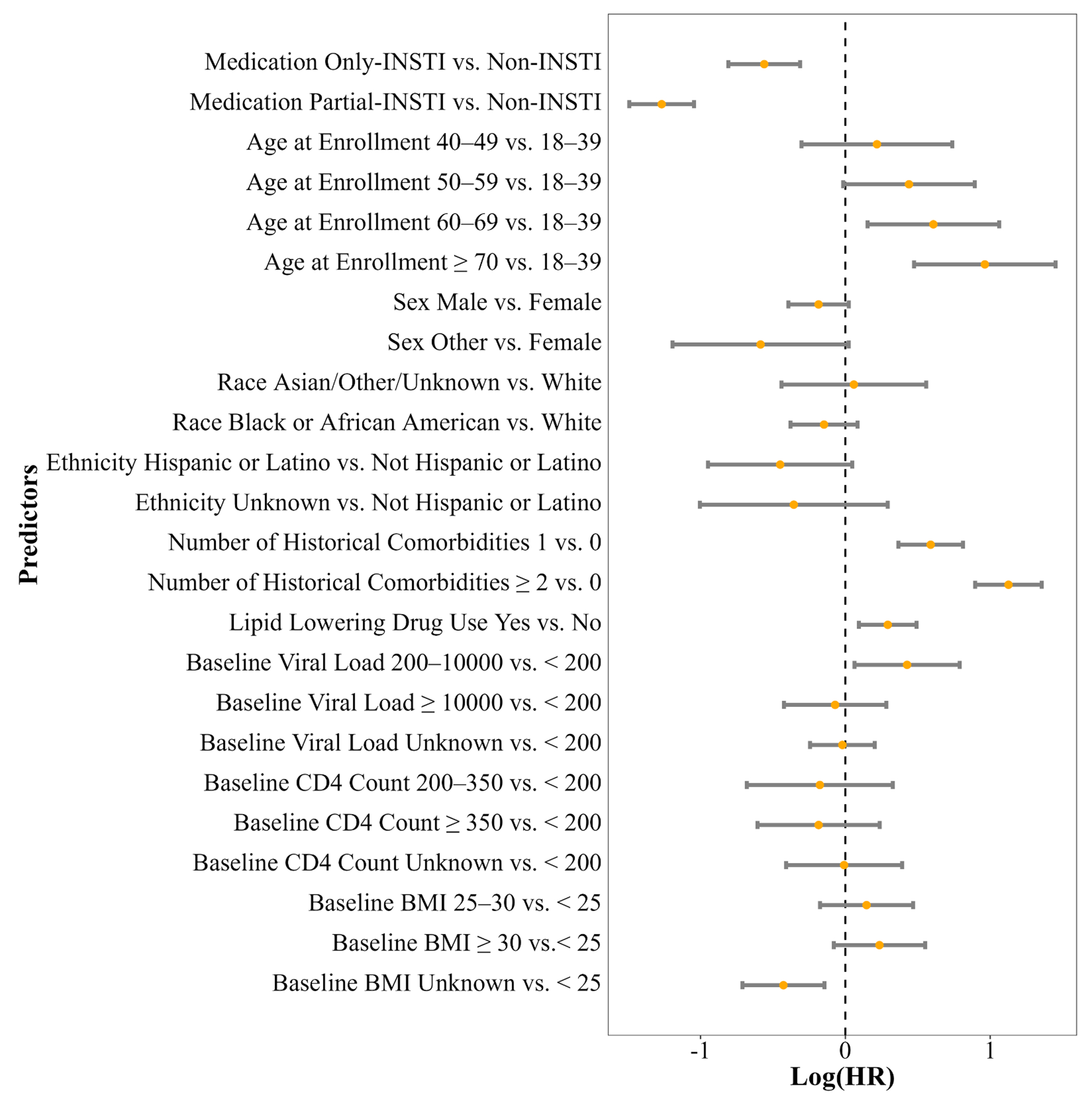
Forest plot for adjusted hazard ratio of the general PWH cohort.

**Fig. 2b.**
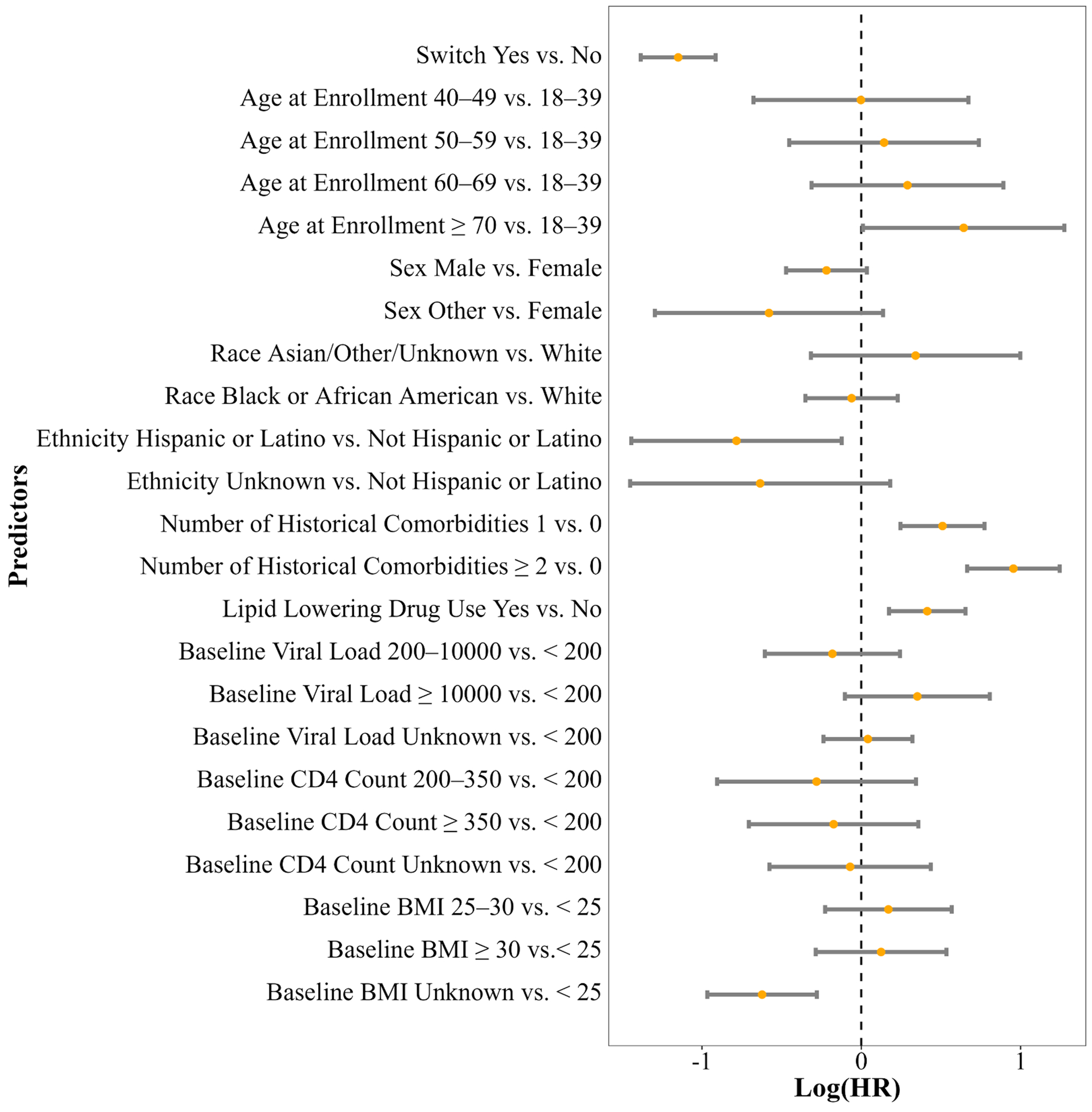
Forest plot for adjusted hazard ratio of the INSTI-naïve cohort.

**Table 1a:** Characteristics distribution by cardiovascular disease for the general PWH cohort.

	Total* (N = 2175)	Cardiovascular disease		p-value^†^
		Censored (N = 1715)	Event (N = 460)	
**ART Regimen Type**				< 0.001
Non-INSTI	437 (20.09)	303 (69.34)	134 (30.66)	
Only-INSTI	783 (36.00)	662 (84.55)	121 (15.45)	
Partial-INSTI	955 (43.91)	750 (78.53)	205 (21.47)	
**Age at Enrollment**	55 (11.74)	54 (11.75)	60 (10.45)	< 0.001
**Age at Enrollment (group)**				< 0.001
18–39^‡^	266 (12.23)	244 (91.73)	22 (8.27)	
40–49	326 (14.99)	285 (87.42)	41 (12.58)	
50–59	757 (34.80)	604 (79.79)	153 (20.21)	
60–69	629 (28.92)	460 (73.13)	169 (26.87)	
≥ 70	197 (9.06)	122 (61.93)	75 (38.07)	
**Sex**				0.048
Female	628 (28.87)	a	a	
Male	1482 (68.14)	1188 (80.16)	294 (19.84)	
Other^§^	65 (2.99)	a	a	
**Race**				0.300
White	508 (23.36)	388 (76.38)	120 (23.62)	
Asian/Other/Unknown^‖^	502 (23.08)	402 (80.08)	100 (19.92)	
Black or African American	1165 (53.56)	925 (79.40)	240 (20.60)	
**Ethnicity**				0.500
Not Hispanic or Latino	1693 (77.84)	1325 (78.26)	368 (21.74)	
Hispanic or Latino	373 (17.15)	302 (80.97)	71 (19.03)	
Unknown	109 (5.01)	88 (80.73)	21 (19.27)	
**Number of Historical Comorbidities**				*<* 0.001
0	1298 (59.68)	1111 (85.59)	187 (14.41)	
1	536 (24.64)	398 (74.25)	138 (25.75)	
≥ 2	341 (15.68)	206 (60.41)	135 (39.59)	
**Lipid Lowering Drug Use**				< 0.001
Yes	862 (39.63)	602 (69.84)	260 (30.16)	
No	1313 (60.37)	1113 (84.77)	200 (15.23)	
**Baseline Viral Load (copies/mL)**				0.300
< 200	761 (34.99)	605 (79.50)	156 (20.50)	
200–10,000	177 (8.14)	135 (76.27)	42 (23.73)	
≥ 10,000	160 (7.36)	118 (73.75)	42 (26.25)	
Unknown	1077 (49.52)	857 (79.57)	220 (20.43)	
**Baseline CD4 Count (cells/μL)**				0.300
< 200	132 (6.07)	103 (78.03)	29 (21.97)	
200–350	150 (6.90)	116 (77.33)	34 (22.67)	
≥ 350	655 (30.11)	534 (81.53)	121 (18.47)	
Unknown	1238 (56.92)	962 (77.71)	276 (22.29)	
**Baseline BMI (kg/m** ^†^ **)**				0.080
< 25	435 (20.00)	359 (82.53)	76 (17.47)	
25–30	392 (18.02)	311 (79.34)	81 (20.66)	
≥ 30	383 (17.61)	288 (75.20)	95 (24.80)	
Unknown	965 (44.37)	757 (78.45)	208 (21.55)	

a Counts less than 20 (and corresponding percentages) cannot be displayed due to NIH All of Us Research Program Data and Statistics Dissemination Policy. Some additional data were collapsed or obscured to prevent secondary calculation of these values.

* N(%); Mean (SD).

† P-values were calculated using Pearson’s Chi-square test for categorical variables and Welch’s two-sample *t*-test for continuous variables.

‡ Age groups 18–29 and 30–39 were combined into a single group 18–39 due to the extremely small size of the subpopulation.

§ The “Other” sex group included participants who responded “Intersex”, “None”, “No matching concept”, “Skip”, and “Prefer not to answer”.

‖ The “Asian/Other/Unknown” race group refer to those who responded “Asian”, “More than one race”, “Other”, “Skip”, and “Prefer not to answer”.

**Table 1b:** Characteristics distribution by cardiovascular disease for the INSTI-naïve cohort.

	Total* (N = 1300)	Cardiovascular disease		p-value^†^
		Censored (N = 980)	Event (N = 320)	
**Switch to INSTI-based Regimen**				< 0.001
Yes	863 (66.38)	677 (78.45)	186 (21.55)	
No	437 (33.62)	303 (69.34)	134 (30.66)	
**Age at Enrollment**	57 (11.12)	56 (11.04)	61 (10.46)	< 0.001
**Age at Enrollment (group)**				< 0.001
18–39^‡^	109 (8.38)	a	a	
40–49	177 (13.62)	a	a	
50–59	473 (36.38)	368 (77.80)	105 (22.20)	
60–69	402 (30.92)	286 (71.14)	116 (28.86)	
≥ 70	139 (10.69)	79 (56.83)	60 (43.17)	
**Sex**				0.300
Female	402 (30.92)	a	a	
Male	858 (66.00)	657 (76.57)	201 (23.43)	
Other^§^	40 (3.08)	a	a	
**Race**				0.400
White	289 (22.23)	210 (72.66)	79 (27.34)	
Asian/Other/Unknown^‖^	290 (22.31)	223 (76.90)	67 (23.10)	
Black or African American	721 (55.46)	547 (75.87)	174 (24.13)	
**Ethnicity**				0.400
Not Hispanic or Latino	1019 (78.38)	760 (74.58)	259 (25.42)	
Hispanic or Latino	215 (16.54)	a	a	
Unknown	66 (5.08)	a	a	
**Number of Historical Comorbidities**				< 0.001
0	807 (62.08)	660 (81.78)	147 (18.22)	
1	325 (25.00)	228 (70.15)	97 (29.85)	
≥ 2	168 (12.92)	92 (54.76)	76 (45.24)	
**Lipid Lowering Drug Use**				< 0.001
Yes	558 (42.92)	369 (66.13)	189 (33.87)	
No	742 (57.08)	611 (82.35)	131 (17.65)	
**Baseline Viral Load (copies/mL)**				0.700
< 200	413 (31.77)	311 (75.30)	102 (24.70)	
200–10,000	113 (8.69)	82 (72.57)	31 (27.43)	
≥ 10,000	99 (7.62)	71 (71.72)	28 (28.28)	
Unknown	675 (51.92)	516 (76.44)	159 (23.56)	
**Baseline CD4 Count (cells/μL)**				> 0.900
< 200	71 (5.46)	a	a	
200–350	93 (7.15)	69 (74.19)	24 (25.81)	
≥ 350	353 (27.15)	268 (75.92)	85 (24.08)	
Unknown	783 (60.23)	a	a	
**Baseline BMI (kg/m** ^†^ **)**				0.400
< 25	219 (16.85)	170 (77.63)	49 (22.37)	
25–30	205 (15.77)	149 (72.68)	56 (27.32)	
≥ 30	188 (14.46)	136 (72.34)	52 (27.66)	
Unknown	688 (52.92)	525 (76.31)	163 (23.69)	

a Counts less than 20 (and corresponding percentages) cannot be displayed due to NIH All of Us Research Program Data and Statistics Dissemination Policy. Some additional data were collapsed or obscured to prevent secondary calculation of these values.

* N(%); Mean (SD).

† P-values were calculated using Pearson’s Chi-square test for categorical variables and Welch’s two-sample *t*-test for continuous variables.

‡ Age groups 18–29 and 30–39 were combined into a single group 18–39 due to the extremely small size of the subpopulation.

§ The “Other” sex group included participants who responded “Intersex”, “None”, “No matching concept”, “Skip”, and “Prefer not to answer”.

‖ The “Asian/Other/Unknown” race group refer to those who responded “Asian”, “More than one race”, “Other”, “Skip”, and “Prefer not to answer”.

**Table 2a:** Factors associated with cardiovascular disease for the general PWH cohort.

	HR^a^ (95 % CI ^b^)	p-value	AHR^c^ (95 % CI)	p-value
**ART Regimen Type (Ref: Non-INSTI)**				
Partial-INSTI	0.36 (0.29, 0.45)	< 0.001	0.34 (0.27, 0.42)	< 0.001
Only-INSTI	0.78 (0.61, 1.01)	0.055	0.65 (0.50, 0.83)	0.001
**Age at Enrollment (Ref: 18–39** ^†^ **)**				
40–49	1.25 (0.74, 2.10)	0.400	1.15 (0.68, 1.95)	0.600
50–59	1.71 (1.09, 2.67)	0.019	1.28 (0.80, 2.03)	0.306
60–69	2.01 (1.29, 3.15)	0.002	1.49 (0.93, 2.38)	0.096
≥ 70	2.59 (1.60, 4.18)	< 0.001	2.05 (1.24, 3.39)	0.005
**Sex (Ref: Female)**				
Male	0.82 (0.68, 1.00)	0.053	0.84 (0.68, 1.04)	0.105
Other^‡^	0.68 (0.38, 1.22)	0.199	0.58 (0.31, 1.07)	0.079
**Race (Ref: White)**				
Asian/Other/Unknown^§^	0.82 (0.63, 1.07)	0.154	1.02 (0.61, 1.68)	0.945
Black or African American	0.88 (0.70, 1.09)	0.242	0.88 (0.69, 1.11)	0.270
**Ethnicity (Ref: Not Hispanic or Latino)**				
Hispanic or Latino	0.83 (0.64, 1.07)	0.156	0.66 (0.40, 1.08)	0.098
Unknown	0.86 (0.55, 1.33)	0.500	0.72 (0.38, 1.39)	0.334
**Number of Historical Comorbidities (Ref: 0)**				
1	1.89 (1.52, 2.36)	< 0.001	1.75 (1.40, 2.20)	< 0.001
≥ 2	3.22 (2.58, 4.02)	< 0.001	3.01 (2.38, 3.79)	< 0.001
**Lipid Lowering Drug Use (Ref: No)**				
Yes	1.53 (1.27, 1.84)	< 0.001	1.34 (1.10, 1.64)	0.004
**Baseline Viral Load (copies/mL) (Ref: < 200)**				
200–10,000	0.78 (0.55, 1.10)	0.158	0.93 (0.65, 1.33)	0.695
≥ 10,000	1.03 (0.73, 1.45)	0.884	1.53 (1.07, 2.20)	0.021
Unknown	0.83 (0.67, 1.02)	0.077	0.98 (0.78, 1.22)	0.857
**Baseline CD4 Count (cells/μL) (Ref: < 200)**				
200–350	0.79 (0.48, 1.30)	0.351	0.84 (0.51, 1.39)	0.494
≥ 350	0.84 (0.56, 1.26)	0.393	0.83 (0.55, 1.27)	0.391
Unknown	0.88 (0.60, 1.28)	0.496	0.99 (0.66, 1.48)	0.966
**Baseline BMI (kg/m^2^) (Ref: < 25)**				
25–30	1.15 (0.84, 1.58)	0.371	1.16 (0.84, 1.60)	0.372
≥ 30	1.59 (1.18, 2.15)	0.003	1.27 (0.92, 1.73)	0.144
Unknown	0.70 (0.54, 0.92)	0.010	0.65 (0.49, 0.87)	0.003

a HR: Hazard ratio;

b CI: Confidence interval;

c AHR: Adjusted hazard ratio.

† Age groups 18–29 and 30–39 were combined into a single group 18–39 due to the extremely small size of the subpopulation.

‡ The “Other” sex group included participants who responded “Intersex”, “None”, “No matching concept”, “Skip”, and “Prefer not to answer”.

§ The “Asian/Other/Unknown” race group refer to those who responded “Asian”, “More than one race”, “Other”, “Skip”, and “Prefer not to answer”.

**Table 2b:** Factors associated with cardiovascular disease for the INSTI-naïve cohort.

	HR^a^ (95 % CI^b^)	p-value	AHR^c^ (95 % CI)	p-value
**Switch to INSTI-based Regimen (Ref: No)**				
Yes	0.33 (0.27, 0.42)	< 0.001	0.32 (0.25, 0.40)	< 0.001
**Age at Enrollment (Ref: 18–39** ^†^ **)**				
40–49	1.09 (0.56, 2.12)	0.800	1.00 (0.51, 1.96)	0.995
50–59	1.50 (0.84, 2.67)	0.170	1.15 (0.64, 2.09)	0.638
60–69	1.71 (0.96, 3.04)	0.068	1.34 (0.73, 2.44)	0.345
≥ 70	2.42 (1.32, 4.42)	0.004	1.90 (1.01, 3.58)	0.046
**Sex (Ref: Female)**				
Male	0.83 (0.65, 1.04)	0.108	0.80 (0.62, 1.04)	0.091
Other^‡^	0.76 (0.38, 1.49)	0.421	0.56 (0.27, 1.15)	0.113
**Race (Ref: White)**				
Asian/Other/Unknown^§^	0.82 (0.59, 1.14)	0.232	1.41 (0.73, 2.71)	0.309
Black or African American	0.92 (0.70, 1.20)	0.529	0.94 (0.70, 1.26)	0.681
**Ethnicity (Ref: Not Hispanic or Latino)**				
Hispanic or Latino	0.76 (0.56, 1.04)	0.091	0.46 (0.24, 0.88)	0.020
Unknown	0.90 (0.53, 1.51)	0.680	0.53 (0.23, 1.20)	0.127
**Number of Historical Comorbidities (Ref: 0)**				
1	1.77 (1.37, 2.28)	< 0.001	1.67 (1.28, 2.17)	< 0.001
≥ 2	2.64 (2.00, 3.48)	< 0.001	2.60 (1.94, 3.47)	< 0.001
**Lipid Lowering Drug Use (Ref: No)**				
Yes	1.61 (1.28, 2.01)	< 0.001	1.51 (1.19, 1.92)	0.001
**Baseline Viral Load (copies/mL) (Ref: < 200)**				
200–10,000	0.70 (0.47, 1.05)	0.085	0.83 (0.55, 1.27)	0.402
≥ 10,000	0.88 (0.57, 1.34)	0.549	1.42 (0.90, 2.24)	0.129
Unknown	0.79 (0.61, 1.02)	0.066	1.04 (0.79, 1.38)	0.771
**Baseline CD4 Count (cells/μL) (Ref: < 200)**				
200–350	0.74 (0.40, 1.36)	0.332	0.76 (0.40, 1.41)	0.378
≥ 350	0.89 (0.54, 1.48)	0.655	0.84 (0.49, 1.43)	0.522
Unknown	0.84 (0.51, 1.36)	0.468	0.93 (0.56, 1.55)	0.787
**Baseline BMI (kg/m^2^) (Ref: < 25)**				
25–30	1.25 (0.86, 1.84)	0.246	1.19 (0.80, 1.76)	0.401
≥ 30	1.45 (0.98, 2.14)	0.063	1.13 (0.75, 1.71)	0.552
Unknown	0.65 (0.47, 0.90)	0.010	0.54 (0.38, 0.76)	< 0.001

a HR: Hazard ratio;

b CI: Confidence interval;

c AHR: Adjusted hazard ratio.

† Age groups 18–29 and 30–39 were combined into a single group 18–39 due to the extremely small size of the subpopulation.

‡ The “Other” sex group included participants who responded “Intersex”, “None”, “No matching concept”, “Skip”, and “Prefer not to answer”.

§ The “Asian/Other/Unknown” race group refer to those who responded “Asian”, “More than one race”, “Other”, “Skip”, and “Prefer not to answer”.

## Data Availability

The data supporting the findings of this study are available through the All of Us Researcher Workbench (https://workbench.researchallofus.org/). Due to privacy and ethical restrictions, the data are not publicly accessible and cannot be shared by the corresponding author upon request. As of the time of publication, access to the All of Us Researcher Workbench is limited to researchers affiliated with institutions that have signed a data use agreement with the All of Us program (https://www.researchallofus.org/register/).

## Abbreviations:

INSTIs: Integrase Strand Transfer Inhibitors
ART: Antiretroviral Therapy
CVD: Cardiovascular Disease
PWH: People with HIV
PIs: Protease Inhibitors
NRTIs: Nucleoside Reverse Transcriptase Inhibitors
AoU: All of Us
EHR: Electronic Health Records
ICD: International Classification of Diseases
NNRTIs: Non-Nucleoside Reverse Transcriptase Inhibitors
BMI: Body Mass Index
SD: Standard Deviation
PH: Proportional Hazards
PSM: Propensity Score Matching
AHR: Adjusted Hazard Ratio
HR: Hazard Ratio
