# Supplementary material for: Impact of integrase strand transfer inhibitors on cardiovascular disease in people with HIV
Source: Ann Epidemiol. Author manuscript; Available in PMC 2026 Jun 30. (PMC13318472; doi:10.1016/j.annepidem.2025.11.006)

**Supplementary Table 1. International Classification of Diseases (ICD) codes used for cardiovascular disease (CVD) identification and screening**

| **Coding Standard** | **Coronary Heart Disease** | **Stroke** |
| --- | --- | --- |
| ICD-9 CM | 410, 411, 412, 413, 414, 429.7, V45.81, V46.82 | 430, 431, 434.0, 434.1, 434.9, 436.X |
| ICD-9 procedure codes | 36.01, 36.02, 36.03, 36.05, 36.09, 36.10, 36.12, 36.13, 36.14, 36.15, 36.16, 36.17, 36.18, 36.19 |  |
| ICD-10 CM | I20.0, I20.1, I20.8, I20.9, I21.09, I21.11, I21.19, I21.29, I21.3, I21.4, I23.0, I24.0, I24.1, I24.8, I25.10, I25.2, I25.3, I25.41, I25.42, I25.5, I25.810, I25.811, I25.812, I25.82, I25.83, I25.84, I25.89, I25.9, I51.0. I51.1, I51.2, I51.3, I51.4, I51.5, I51.7, I51.81, I51.89, I51.9, I97.0, I97.110, I97.130, I97.190, Z95.1, Z98.61 | I60, I60.0, I60.1, I60.2, I60.3, I60.4, I60.5, I60.6, I60.7, I60.8, I60.9, I61, I61.0 , I61.1, I61.2, I61.3, I61.4, I61.5, I61.6, I61.8, I61.9, I63, I63.0, I63.1, I63.2, I63.3, I63.4, I63.5, I63.6, I63.8, I63.9, I63.X, I64, I64.X |
| ICD-10 procedure codes | 0210093, 0210098, 0210099, 021009C, 021009F, 021009W, 02100A3, 02100A8, 02100A9, 02100AC, 02100AF, 02100AW, 02100J3, 02100J8, 02100J9, 02100JC, 02100JF, 02100JW, 02100K3, 02100K8, 02100K9, 02100KC, 02100KF, 02100KW, 02100Z3, 02100Z8, 02100Z9, 02100ZC, 02100ZF, 0210493, 0210498, 0210499, 021049C, 021049F, 021049W, 02104A3, 02104A8, 02104A9, 02104AC, 02104AF, 02104AW, 02104J3, 02104J8, 02104J9, 02104JC, 02104JF, 02104JW, 02104K3, 02104K8, 02104K9, 02104KC, 02104KF, 02104KW, 02104Z3, 02104Z8, 02104Z9, 02104ZC, 02104ZF, 0211098, 0211099, 021109C, 021109W, 02110A8, 02110A9, 02110AC, 02110AW, 02110J8, 02110J9, 02110JC, 02110JW, 02110K8, 02110K9, 02110KC, 02110KW, 02110Z8, 02110Z9, 02110ZC, 0211498, 0211499, 021149C, 021149W, 02114A8, 02114A9, 02114AC, 02114AW, 02114J8, 02114J9, 02114JC, 02114JW, 02114K8, 02114K9, 02114KC, 02114KW, 02114Z8, 02114Z9, 02114ZC, 021209C, 021209W, 02120AC, 02120AW, 02120JC, 02120JW, 02120KC, 02120KW, 02120ZC, 021249C, 021249W, 02124AC, 02124AW, 02124JC, 02124JW, 02124KC, 02124KW, 02124ZC, 021309C, 021309W, 02130AC, 02130AW, 02130JC, 02130JW, 02130KC, 02130KW, 02130ZC, 021349C, 021349W, 02134AC, 02134AW, 02134JC, 02134JW, 02134KC, 02134KW, 02134ZC, 02700ZZ, 02710ZZ, 02720ZZ, 02730ZZ, 02C00ZZ, 02C03ZZ, 02C04ZZ, 02C10ZZ, 02C13ZZ, 02C14ZZ, 02C20ZZ, 02C23ZZ, 02C24ZZ, 02C30ZZ, 02C33ZZ, 02C34ZZ |  |

**Supplementary Table 2. Antiretroviral therapy (ART) related RxNorm codes and corresponding medication labels used for data extraction**

See the separate word file “supplementary table 2.docx”.

**Supplementary Table 3. Classification of the five main classes of ART**

| **ART Classes** | **Generic names** |
| --- | --- |
| PI | amprenavir, atazanavir, atazanavir sulfate, darunavir, fosamprenavir, indinavir, lopinavir, nelfinavir, ritonavir, saquinavir, tipranavir |
| NRTI | abacavir, abacavir sulfate, didanosine, emtricitabine, lamivudine, stavudine, tenofovir alafenamide, tenofovir DF, zalcitabine, zidovudine, tenofovir disoproxil fumarate |
| NNRTI | delavirdine, doravirine, efavirenz, etravirine, nevirapine, rilpivirine, dasabuvir |
| INSTI | bictegravir, cabotegravir, dolutegravir, elvitegravir, raltegravir |
| OTHERS | maraviroc, ibalizumab, ibalizumab-uiyk, cobicistat, fostemsavir, lenacapavir |

**Supplementary Table 4. Data examples of INSTI exposure cases**

| **Cohort** | **INSTI exposure type** | **Simulated time indicator (years since ART initiation)** | | | |
| --- | --- | --- | --- | --- | --- |
|  |  | **0-3** | **4-6** | **7-9** | **10-11** |
| INSTI-naïve cohort | Switch to INSTI: No | NRTI+PI | NRTI+NNRTI | NRTI+PI | NRTI+PI |
|  | Switch to INSTI: Yes | NRTI+PI | NRTI+INSTI | NRTI+INSTI | NNRTI+NRTI+INSTI |
| General PWH cohort | Non-INSTI | NNRTI+PI+OTHER | NNRTI+PI | PI+OTHER | NNRTI+NRTI |
|  | Only-INSTI | NRTI+INSTI | NNRTI+INSTI | NNRTI+INSTI | NNRTI+INSTI |
|  | Partial-INSTI | NRTI+INSTI | NRTI+INSTI | NNRTI+PI | NNRTI+NRTI |
|  |  | NRTI+NNRTI | PI+INSTI | NRTI+INSTI | NRTI+INSTI |
|  |  | NRTI+INSTI | NRTI+INSTI | NNRTI+NRTI | PI+INSTI |
|  |  | NRTI+NNRTI | NRTI+INSTI+PI | NNRTI+PI | PI+INSTI |

**Supplementary Table 5. Concept IDs used for identifying the lipid-lowering drug**

| **Concept IDs** | 1592085, 40165636, 1549686, 1510813, 1551860, 1545958, 1539403 |
| --- | --- |

**Supplementary Table 6. ICD codes used for identifying historical comorbidities**

| **Name** | **ICD-9/10 Code** |
| --- | --- |
| Cancer | C00.%, C01.%, C02.%, C03.%, C04.%, C05.%, C06.%, C07.%, C08.%, C09.%, C10.%, C11.%, C12.%, C13.%, C14.%, C15.%, C16.%, C17.%, C18.%, C19.%, C20.%, C21.%, C22.%, C23.%, C24.%, C25.%, C26.%, C30.%, C31.%, C32.%, C33.%, C34.%, C37.%, C38.%, C39.%, C40.%, C41.%, C43.%, C45.%, C46.%, C47.%, C48.%, C60.%, C61.%, C62.%, C63.%, C64.%, C65.%, C66.%, C67.%, C68.%, C69.%, C70.%, C71.%, C72.%, C73.%, C74.%, C75.%, C76.%, C81.%, C82.%, C83.%, C84.%, C85.%, C88.%, C90.%, C91.%, C92.%, C93.%, C94.%, C95.%, C96.%, C97.%, 140.%, 141.%, 142.%, 143.%, 144.%, 145.%, 146.%, 147.%, 148.%, 149.%, 150.%, 151.%, 152.%, 153.%, 154.%, 155.%, 156.%, 157.%, 158.%, 159.%, 160.%, 161.%, 162.%, 163.%, 164.%, 165.%, 166.%, 167.%, 168.%, 169.%, 170.%, 171.%, 172.%, 174.%, 175.%, 176.%, 177.%, 178.%, 179.%, 180.%, 181.%, 182.%, 183.%, 184.%, 185.%, 186.%, 187.%, 188.%, 189.%, 190.%, 191.%, 192.%, 193.%, 194.%, 195.%, 200.%, 201.%, 202.%, 203.%, 204.%, 205.%, 206.%, 207.%, 208.%, 238.6, C77.%, C78.%, C79.%, C80.%, 196.%, 197.%, 198.%, 199.% |
| Chronic Obstructive Pulmonary Disease | I27.8, I27.9, J40.%, J41.%, J42.%, J43.%, J44.%, J45.%, J46.%, J47.%, J60.%, J61.%, J62.%, J63.%, J64.%, J65.%, J66.%, J67.%, J68.4, J70.1, J70.3, 416.8, 416.9, 490.%, 491.%, 492.%, 493.%, 494.%, 495.%, 496.%, 497.%, 498.%, 499.%, 500.%, 501.%, 502.%, 503.%, 504.%, 505.%, 506.4, 508.1, 508.8 |
| Dementia | F00.%, F03.%, F05.1, G30.%, G31.1, 290.%, 294.1, 331.2 |
| Diabetes | E10.0, E10.1, E10.6, E10.8, E10.9, E11.0, E11.1, E11.6, E11.8, E11.9, E12.0, E12.1, E12.6, E12.8, E12.9, E13.0, E13.1, E13.6, E13.8, E13.9, E14.0, E14.1, E14.6, E14.8, E14.9, 250.0, 250.1, 250.2, 250.3, 250.8, 250.9, 250.7, E10.2, E10.3, E10.4, E10.5, E10.7, E11.2, E11.3, E11.4, E11.5, E11.7, E12.2, E12.3, E12.4, E12.5, E12.7, E13.2, E13.3, E13.4, E13.5, E13.7, E14.2, E14.3, E14.4, E14.5, E14.7, 250.4, 250.5, 250.6, 250.7 |
| Hemiplegia/Paraplegia | G04.1, G11.4, G80.1, G80.2, G81.%, G82.%, G83.0, G83.1, G83.2, G83.3, G83.4, G83.9, 334.1, 342.%, 343.%, 344.0, 344.1, 344.2, 344.3, 344.4, 344.5, 344.6, 344.9 |
| Liver Disease | B18.%, K70.0, K70.1, K70.2, K70.3, K70.9, K71.3, K71.4, K71.5, K71.7, K73.%, K74.%, K76.0, K76.2, K76.3, K76.4, K76.8, K76.9, Z94.4, 070.22, 070.23, 070.32, 070.33, 070.44, 070.54, 070.6, 070.9, 570.%, 571.%, 573.3, 573.4, 573.8, 573.9, V42.7, I85.0, I85.9, I86.4, I98.2, K70.4, K71.1, K72.1, K72.9, K76.5, K76.6, K76.7, 456.0, 456.1, 456.2, 572.2, 572.3, 572.4, 572.5, 572.6, 572.7, 572.8 |
| Peptic Ulcer Disease | K25.%, K26.%, K27.%, K28.%, 531.%, 532.%, 533.%, 534.% |
| Renal Disease | I12.0, I13.1, N03.2, N03.3, N03.4, N03.5, N03.6, N03.7, N05.2, N05.3, N05.4, N05.5, N05.6, N05.7, N18.%, N19.%, N25.0, Z49.0, Z49.1, Z49.2, Z94.0, Z99.2, 403.01, 403.11, 403.91, 404.02, 404.03, 404.12, 404.13, 404.92, 404.93, 582.%, 583.0, 583.1, 583.2, 583.3, 583.4, 583.5, 583.6, 583.7, 585.%, 586.%, V42.0, V45.1, V56.%, 588.% |
| Rheumatic Disease | M05.%, M06.%, M31.5, M32.%, M33.%, M34.%, M35.1, M35.3, M36.0, 446.5, 710.0, 710.1, 710.2, 710.3, 710.4, 714.0, 714.1, 714.2, 714.8, 725.%, 714.81 |

**Supplementary Table 7. Factors associated with CVD in the sensitivity analysis restricting the partial-INSTI group to individuals with a single switch to INSTI**

|  | **AHR^1^ (95 % CI^2^)** | **p-value** |
| --- | --- | --- |
| **ART Regimen Type (Ref: Non-INSTI)** |  |  |
| Switch-INSTI | 0.33 (0.26, 0.42) | < 0.001 |
| Only-INSTI | 0.65 (0.50, 0.83) | 0.001 |
| **Age at Enrollment (Ref: 18–39^3^)** |  |  |
| 40–49 | 1.24 (0.70, 2.18) | 0.464 |
| 50–59 | 1.34 (0.81, 2.21) | 0.250 |
| 60–69 | 1.58 (0.96, 2.62) | 0.074 |
| ≥ 70 | 2.18 (1.27, 3.74) | 0.005 |
| **Sex (Ref: Female)** |  |  |
| Male | 0.81 (0.65, 1.01) | 0.063 |
| Other^4^ | 0.53 (0.27, 1.03) | 0.060 |
| **Race (Ref: White)** |  |  |
| Asian/Other/Unknown^5^ | 0.72 (0.41, 1.29) | 0.275 |
| Black or African American | 0.87 (0.68, 1.11) | 0.270 |
| **Ethnicity (Ref: Not Hispanic or Latino)** |  |  |
| Hispanic or Latino | 0.89 (0.50, 1.58) | 0.691 |
| Unknown | 0.91 (0.43, 1.91) | 0.801 |
| **Number of Historical Comorbidities (Ref: 0)** |  |  |
| 1 | 1.80 (1.42, 2.28) | < 0.001 |
| ≥ 2 | 3.05 (2.38, 3.92) | < 0.001 |
| **Lipid Lowering Drug Use (Ref: No)** |  |  |
| Yes | 1.40 (1.14, 1.73) | 0.002 |
| **Baseline Viral Load (copies/mL) (Ref: < 200)** |  |  |
| 200–10000 | 1.06 (0.73, 1.54) | 0.769 |
| ≥ 10000 | 1.66 (1.13, 2.46) | 0.010 |
| Unknown | 1.02 (0.81, 1.30) | 0.840 |
| **Baseline CD4 Count (cells/µL) (Ref: < 200)** |  |  |
| 200–350 | 0.83 (0.48, 1.43) | 0.506 |
| ≥ 350 | 0.85 (0.55, 1.32) | 0.476 |
| Unknown | 1.02 (0.67, 1.55) | 0.939 |
| **Baseline BMI (kg/m^2^) (Ref: < 25)** |  |  |
| 25–30 | 1.14 (0.82, 1.60) | 0.435 |
| ≥ 30 | 1.20 (0.86, 1.68) | 0.277 |
| Unknown | 0.63 (0.47, 0.85) | 0.002 |

^1^AHR: Adjusted hazard ratio; ^2^CI: Confidence interval.

^3^Age groups 18–29 and 30–39 were combined into a single group 18–39 due to the extremely small size of the subpopulation.

^4^The “Other” sex group included participants who responded “Intersex”, “None”, “No matching concept”, “Skip”, and “Prefer not to answer”.

^5^The “Asian/Other/Unknown” race group refer to those who responded “Asian”, “More than one race”, “Other”, “Skip”, and “Prefer not to answer”.

**Supplementary Table 8. Factors associated with CVD in the sensitivity analysis using years of INSTI-based ART use as a predictor**

|  | **AHR^1^ (95 % CI^2^)** | **p-value** |
| --- | --- | --- |
| **Time on INSTI-based regimen (year)** | 0.80 (0.77, 0.83) | < 0.001 |
| **Age at Enrollment (Ref: 18–39^3^)** |  |  |
| 40–49 | 0.96 (0.57, 1.63) | 0.890 |
| 50–59 | 1.20 (0.76, 1.91) | 0.432 |
| 60–69 | 1.49 (0.93, 2.39) | 0.094 |
| ≥70 | 1.83 (1.10, 3.03) | 0.020 |
| **Sex (Ref: Female)** |  |  |
| Male | 0.87 (0.70, 1.07) | 0.190 |
| Other^4^ | 0.70 (0.38, 1.29) | 0.250 |
| **Race (Ref: White)** |  |  |
| Asian/Other/Unknown^5^ | 1.10 (0.66, 1.82) | 0.726 |
| Black or African American | 0.84 (0.67, 1.07) | 0.162 |
| **Ethnicity (Ref: Not Hispanic or Latino)** |  |  |
| Hispanic or Latino | 0.64 (0.39, 1.06) | 0.085 |
| Unknown | 0.65 (0.34, 1.25) | 0.195 |
| **Number of Historical Comorbidities (Ref: 0)** |  |  |
| 1 | 1.79 (1.43, 2.24) | < 0.001 |
| ≥ 2 | 3.04 (2.41, 3.83) | < 0.001 |
| **Lipid Lowering Drug Use (Ref: No)** |  |  |
| Yes | 1.41 (1.16, 1.73) | 0.001 |
| **Baseline Viral Load (copies/mL) (Ref: < 200)** |  |  |
| 200–10000 | 0.81 (0.57, 1.16) | 0.255 |
| ≥ 10000 | 1.36 (0.93, 1.99) | 0.107 |
| Unknown | 0.89 (0.71, 1.12) | 0.321 |
| **Baseline CD4 Count (cells/µL) (Ref: < 200)** |  |  |
| 200–350 | 0.84 (0.50, 1.40) | 0.499 |
| ≥ 350 | 0.79 (0.51, 1.22) | 0.281 |
| Unknown | 0.94 (0.62, 1.43) | 0.789 |
| **Baseline BMI (kg/m^2^) (Ref: < 25)** |  |  |
| 25–30 | 1.25 (0.91, 1.73) | 0.172 |
| ≥ 30 | 1.39 (1.01, 1.92) | 0.042 |
| Unknown | 0.67 (0.51, 0.89) | 0.006 |

^1^AHR: Adjusted hazard ratio; ^2^CI: Confidence interval.

^3^Age groups 18–29 and 30–39 were combined into a single group 18–39 due to the extremely small size of the subpopulation.

^4^The “Other” sex group included participants who responded “Intersex”, “None”, “No matching concept”, “Skip”, and “Prefer not to answer”.

^5^The “Asian/Other/Unknown” race group refer to those who responded “Asian”, “More than one race”, “Other”, “Skip”, and “Prefer not to answer”.

**Supplementary Table 9. Characteristics distribution by INSTI exposure for the INSTI-naïve cohort**

|  | **Total (N = 1300)^1^** | **Switch to INSTI-based Regimen** | | **p-value^2^** |
| --- | --- | --- | --- | --- |
|  |  | **No (N = 437)** | **Yes (N = 863)** |  |
| **Survival Status** |  |  |  | < 0.001 |
| Experienced CVD | 320 (24.62) | 134 (41.88) | 186 (58.13) |  |
| Censored | 980 (75.38) | 303 (30.92) | 677 (69.08) |  |
| **Age at Enrollment (group)** |  |  |  | 0.600 |
| 18–39^3^ | 109 (8.38) | 38 (34.86) | 71 (65.14) |  |
| 40–49 | 177 (13.62) | 50 (28.25) | 127 (71.75) |  |
| 50–59 | 473 (36.38) | 162 (34.25) | 311 (65.75) |  |
| 60–69 | 402 (30.92) | 138 (34.33) | 264 (65.67) |  |
| ≥ 70 | 139 (10.69) | 49 (35.25) | 90 (64.75) |  |
| **Sex** |  |  |  | 0.300 |
| Female | 402 (30.92) | * | * |  |
| Male | 858 (66.00) | 281 (32.75) | 577 (67.25) |  |
| Other^4^ | 40 (3.08) | * | * |  |
| **Race** |  |  |  | > 0.900 |
| White | 289 (22.23) | 94 (32.53) | 195 (67.47) |  |
| Asian/Other/Unknown^5^ | 290 (22.31) | 98 (33.79) | 192 (66.21) |  |
| Black or African American | 721 (55.46) | 245 (33.98) | 476 (66.02) |  |
| **Ethnicity** |  |  |  | 0.800 |
| Not Hispanic or Latino | 1,019 (78.38) | 338 (33.17) | 681 (66.83) |  |
| Hispanic or Latino | 215 (16.54) | 75 (34.88) | 140 (65.12) |  |
| Unknown | 66 (5.08) | 24 (36.36) | 42 (63.64) |  |
| **Number of Historical Comorbidities** |  |  |  | 0.700 |
| 0 | 807 (62.08) | 264 (32.71) | 543 (67.29) |  |
| 1 | 325 (25.00) | 113 (34.77) | 212 (65.23) |  |
| ≥ 2 | 168 (12.92) | 60 (35.71) | 108 (64.29) |  |
| **Lipid Lowering Drug Use** |  |  |  | 0.300 |
| Yes | 558 (42.92) | 178 (31.90) | 380 (68.10) |  |
| No | 742 (57.08) | 259 (34.91) | 483 (65.09) |  |
| **Baseline Viral Load (copies/mL)** |  |  |  | 0.040 |
| < 200 | 413 (31.77) | 140 (33.90) | 273 (66.10) |  |
| 200–10000 | 113 (8.69) | 27 (23.89) | 86 (76.11) |  |
| ≥ 10000 | 99 (7.62) | 27 (27.27) | 72 (72.73) |  |
| Unknown | 675 (51.92) | 243 (36.00) | 432 (64.00) |  |
| **Baseline CD4 Count (cells/µL)** |  |  |  | 0.034 |
| < 200 | 71 (5.46) | * | * |  |
| 200–350 | 93 (7.15) | 22 (23.66) | 71 (76.34) |  |
| ≥ 350 | 353 (27.15) | 112 (31.73) | 241 (68.27) |  |
| Unknown | 783 (60.23) | * | * |  |
| **Baseline BMI (kg/m^2^)** |  |  |  | 0.200 |
| < 25 | 219 (16.85) | 61 (27.85) | 158 (72.15) |  |
| 25–30 | 205 (15.77) | 70 (34.15) | 135 (65.85) |  |
| ≥ 30 | 188 (14.46) | 71 (37.77) | 117 (62.23) |  |
| Unknown | 688 (52.92) | 235 (34.16) | 453 (65.84) |  |

^1^N (%).

^2^P-values were calculated using Pearson’s Chi-square test for categorical variables and Welch’s two-sample t-test for continuous variables.

^3^Age groups 18–29 and 30–39 were combined into a single group 18–39 due to the extremely small size of the subpopulation.

^4^The “Other” sex group included participants who responded “Intersex”, “None”, “No matching concept”, “Skip”, and “Prefer not to answer”.

^5^The “Asian/Other/Unknown” race group refer to those who responded “Asian”, “More than one race”, “Other”, “Skip”, and “Prefer not to answer”.

*Counts less than 20 (and corresponding percentages) cannot be displayed due to NIH All of Us Research Program Data and Statistics Dissemination Policy. Some additional data were collapsed or obscured to prevent secondary calculation of these values.

**Supplementary Table 10. Characteristics distribution by INSTI exposure balanced by PSM for the INSTI-naïve cohort**

|  | **Total (N = 824)^1^** | **Switch to INSTI-based Regimen** | | **p-value^2^** |
| --- | --- | --- | --- | --- |
|  |  | **No (N = 412)** | **Yes (N = 412)** |  |
| **Survival Status** |  |  |  | < 0.001 |
| Experienced CVD | 197 (23.91) | 122 (61.93) | 75 (38.07) |  |
| Censored | 627 (76.09) | 290 (46.25) | 337 (53.75) |  |
| **Age at Enrollment (group)** |  |  |  | 0.500 |
| 18–39^3^ | 70 (8.50) | 35 (50.00) | 35 (50.00) |  |
| 40–49 | 109 (13.23) | 47 (43.12) | 62 (56.88) |  |
| 50–59 | 306 (37.14) | 154 (50.33) | 152 (49.67) |  |
| 60–69 | 256 (31.07) | 129 (50.39) | 127 (49.61) |  |
| ≥ 70 | 83 (10.07) | 47 (56.63) | 36 (43.37) |  |
| **Sex** |  |  |  | 0.600 |
| Female | 258 (31.31) | * | * |  |
| Male | 539 (65.41) | 268 (49.72) | 271 (50.28) |  |
| Other^4^ | 27 (3.28) | * | * |  |
| **Race** |  |  |  | 0.700 |
| White | 192 (23.30) | 91 (47.40) | 101 (52.60) |  |
| Asian/Other/Unknown^5^ | 178 (21.60) | 90 (50.56) | 88 (49.44) |  |
| Black or African American | 454 (55.10) | 231 (50.88) | 223 (49.12) |  |
| **Ethnicity** |  |  |  | 0.700 |
| Not Hispanic or Latino | 648 (78.64) | 320 (49.38) | 328 (50.62) |  |
| Hispanic or Latino | 134 (16.26) | 71 (52.99) | 63 (47.01) |  |
| Unknown | 42 (5.10) | 21 (50.00) | 21 (50.00) |  |
| **Number of Historical Comorbidities** |  |  |  | 0.500 |
| 0 | 526 (63.83) | 255 (48.48) | 271 (51.52) |  |
| 1 | 199 (24.15) | 105 (52.76) | 94 (47.24) |  |
| ≥ 2 | 99 (12.01) | 52 (52.53) | 47 (47.47) |  |
| **Lipid Lowering Drug Use** |  |  |  | 0.120 |
| Yes | 329 (39.93) | 176 (53.50) | 153 (46.50) |  |
| No | 495 (60.07) | 236 (47.68) | 259 (52.32) |  |
| **Baseline Viral Load (copies/mL)** |  |  |  | 0.700 |
| < 200 | 263 (31.92) | 132 (50.19) | 131 (49.81) |  |
| 200–10000 | 44 (5.34) | 24 (54.55) | 20 (45.45) |  |
| ≥ 10000 | 55 (6.67) | 24 (43.64) | 31 (56.36) |  |
| Unknown | 462 (56.07) | 232 (50.22) | 230 (49.78) |  |
| **Baseline CD4 Count (cells/µL)** |  |  |  | 0.800 |
| < 200 | 30 (3.64) | * | * |  |
| 200–350 | 37 (4.49) | * | * |  |
| ≥ 350 | 209 (25.36) | 108 (51.67) | 101 (48.33) |  |
| Unknown | 548 (66.50) | 268 (48.91) | 280 (51.09) |  |
| **Baseline BMI (kg/m^2^)** |  |  |  | > 0.900 |
| < 25 | 119 (14.44) | 59 (49.58) | 60 (50.42) |  |
| 25–30 | 126 (15.29) | 66 (52.38) | 60 (47.62) |  |
| ≥ 30 | 112 (13.59) | 56 (50.00) | 56 (50.00) |  |
| Unknown | 467 (56.67) | 231 (49.46) | 236 (50.54) |  |

^1^N (%).

^2^P-values were calculated using Pearson’s Chi-square test for categorical variables and Welch’s two-sample t-test for continuous variables.

^3^Age groups 18–29 and 30–39 were combined into a single group 18–39 due to the extremely small size of the subpopulation.

^4^The “Other” sex group included participants who responded “Intersex”, “None”, “No matching concept”, “Skip”, and “Prefer not to answer”.

^5^The “Asian/Other/Unknown” race group refer to those who responded “Asian”, “More than one race”, “Other”, “Skip”, and “Prefer not to answer”.

*Counts less than 20 (and corresponding percentages) cannot be displayed due to NIH All of Us Research Program Data and Statistics Dissemination Policy. Some additional data were collapsed or obscured to prevent secondary calculation of these values.

**Supplementary Table 11. Characteristics distribution by INSTI exposure for the general PWH cohort**

|  | **Total (N = 2175)^1^** | **ART Regimen Type** | | | **p-value^2^** |
| --- | --- | --- | --- | --- | --- |
|  |  | **Non-INSTI (N = 437)** | **Only-INSTI (N = 783)** | **Partial-INSTI (N = 955)** |  |
| **Survival Status** |  |  |  |  | < 0.001 |
| Experienced CVD | 460 (21.15) | 134 (29.13) | 121 (26.30) | 205 (44.57) |  |
| Censored | 1715 (78.85) | 303 (17.67) | 662 (38.60) | 750 (43.73) |  |
| **Age at Enrollment (group)** |  |  |  |  | < 0.001 |
| 18–39^3^ | 266 (12.23) | 38 (14.29) | 140 (52.63) | 88 (33.08) |  |
| 40–49 | 326 (14.99) | 50 (15.34) | 135 (41.41) | 141 (43.25) |  |
| 50–59 | 757 (34.80) | 162 (21.40) | 247 (32.63) | 348 (45.97) |  |
| 60–69 | 629 (28.92) | 138 (21.94) | 208 (33.07) | 283 (44.99) |  |
| ≥ 70 | 197 (9.06) | 49 (24.87) | 53 (26.90) | 95 (48.22) |  |
| **Sex** |  |  |  |  | 0.086 |
| Female | 628 (28.87) | * | * | 286 (45.54) |  |
| Male | 1482 (68.14) | 281 (18.96) | 558 (37.65) | 643 (43.39) |  |
| Other^4^ | 65 (2.99) | * | * | 26 (40.00) |  |
| **Race** |  |  |  |  | 0.200 |
| White | 508 (23.36) | 94 (18.50) | 198 (38.98) | 216 (42.52) |  |
| Asian/Other/Unknown^5^ | 502 (23.08) | 98 (19.52) | 193 (38.45) | 211 (42.03) |  |
| Black or African American | 1165 (53.56) | 245 (21.03) | 392 (33.65) | 528 (45.32) |  |
| **Ethnicity** |  |  |  |  | 0.600 |
| Not Hispanic or Latino | 1693 (77.84) | 338 (19.96) | 600 (35.44) | 755 (44.60) |  |
| Hispanic or Latino | 373 (17.15) | 75 (20.11) | 147 (39.41) | 151 (40.48) |  |
| Unknown | 109 (5.01) | 24 (22.02) | 36 (33.03) | 49 (44.95) |  |
| **Number of Historical Comorbidities** |  |  |  |  | 0.055 |
| 0 | 1298 (59.68) | 264 (20.34) | 447 (34.44) | 587 (45.22) |  |
| 1 | 536 (24.64) | 113 (21.08) | 189 (35.26) | 234 (43.66) |  |
| ≥ 2 | 341 (15.68) | 60 (17.60) | 147 (43.11) | 134 (39.30) |  |
| **Lipid Lowering Drug Use** |  |  |  |  | 0.002 |
| Yes | 862 (39.63) | 178 (20.65) | 273 (31.67) | 411 (47.68) |  |
| No | 1313 (60.37) | 259 (19.73) | 510 (38.84) | 544 (41.43) |  |
| **Baseline Viral Load (copies/mL)** |  |  |  |  | < 0.001 |
| < 200 | 761 (34.99) | 140 (18.40) | 314 (41.26) | 307 (40.34) |  |
| 200–10000 | 177 (8.14) | 27 (15.25) | 57 (32.20) | 93 (52.54) |  |
| ≥ 10000 | 160 (7.36) | 27 (16.88) | 50 (31.25) | 83 (51.88) |  |
| Unknown | 1077 (49.52) | 243 (22.56) | 362 (33.61) | 472 (43.83) |  |
| **Baseline CD4 Count (cells/µL)** |  |  |  |  | < 0.001 |
| < 200 | 132 (6.07) | * | * | 62 (46.97) |  |
| 200–350 | 150 (6.90) | 22 (14.67) | 47 (31.33) | 81 (54.00) |  |
| ≥ 350 | 655 (30.11) | 112 (17.10) | 289 (44.12) | 254 (38.78) |  |
| Unknown | 1238 (56.92) | * | * | 558 (45.07) |  |
| **Baseline BMI (kg/m^2^)** |  |  |  |  | < 0.001 |
| < 25 | 435 (20.00) | 61 (14.02) | 196 (45.06) | 178 (40.92) |  |
| 25–30 | 392 (18.02) | 70 (17.86) | 169 (43.11) | 153 (39.03) |  |
| ≥ 30 | 383 (17.61) | 71 (18.54) | 174 (45.43) | 138 (36.03) |  |
| Unknown | 965 (44.37) | 235 (24.35) | 244 (25.28) | 486 (50.36) |  |

^1^N (%).

^2^P-values were calculated using Pearson’s Chi-square test for categorical variables and Welch’s two-sample t-test for continuous variables.

^3^Age groups 18–29 and 30–39 were combined into a single group 18–39 due to the extremely small size of the subpopulation.

^4^The “Other” sex group included participants who responded “Intersex”, “None”, “No matching concept”, “Skip”, and “Prefer not to answer”.

^5^The “Asian/Other/Unknown” race group refer to those who responded “Asian”, “More than one race”, “Other”, “Skip”, and “Prefer not to answer”.

*Counts less than 20 (and corresponding percentages) cannot be displayed due to NIH All of Us Research Program Data and Statistics Dissemination Policy. Some additional data were collapsed or obscured to prevent secondary calculation of these values.

**Supplementary Table 12. Characteristics distribution by INSTI exposure balanced by PSM for the general PWH cohort**

|  | **Total (N = 1218)^1^** | **ART Regimen Type** | | | **p-value^2^** |
| --- | --- | --- | --- | --- | --- |
|  |  | **Non-INSTI (N = 429)** | **Only-INSTI (N = 368)** | **Partial-INSTI (N = 421)** |  |
| **Survival Status** |  |  |  |  | < 0.001 |
| Experienced CVD | 268 (22.00) | 131 (48.88) | 53 (19.78) | 84 (31.34) |  |
| Censored | 950 (78.00) | 298 (31.37) | 315 (33.16) | 337 (35.47) |  |
| **Age at Enrollment (group)** |  |  |  |  | > 0.900 |
| 18–39^3^ | 112 (9.20) | 38 (33.93) | 37 (33.04) | 37 (33.04) |  |
| 40–49 | 157 (12.89) | 50 (31.85) | 52 (33.12) | 55 (35.03) |  |
| 50–59 | 455 (37.36) | 157 (34.51) | 137 (30.11) | 161 (35.38) |  |
| 60–69 | 381 (31.28) | 138 (36.22) | 113 (29.66) | 130 (34.12) |  |
| ≥ 70 | 113 (9.28) | 46 (40.71) | 29 (25.66) | 38 (33.63) |  |
| **Sex** |  |  |  |  | 0.092 |
| Female | 363 (29.80) | * | * | * |  |
| Male | 819 (67.24) | 277 (33.82) | 264 (32.23) | 278 (33.94) |  |
| Other^4^ | 36 (2.96) | * | * | * |  |
| **Race** |  |  |  |  | 0.600 |
| White | 280 (22.99) | 91 (32.50) | 90 (32.14) | 99 (35.36) |  |
| Asian/Other/Unknown^5^ | 286 (23.48) | 96 (33.57) | 92 (32.17) | 98 (34.27) |  |
| Black or African American | 652 (53.53) | 242 (37.12) | 186 (28.53) | 224 (34.36) |  |
| **Ethnicity** |  |  |  |  | > 0.900 |
| Not Hispanic or Latino | 941 (77.26) | 331 (35.18) | 282 (29.97) | 328 (34.86) |  |
| Hispanic or Latino | 214 (17.57) | 74 (34.58) | * | * |  |
| Unknown | 63 (5.17) | 24 (38.10) | * | * |  |
| **Number of Historical Comorbidities** |  |  |  |  | 0.600 |
| 0 | 752 (61.74) | 264 (35.11) | 217 (28.86) | 271 (36.04) |  |
| 1 | 294 (24.14) | 105 (35.71) | 97 (32.99) | 92 (31.29) |  |
| ≥ 2 | 172 (14.12) | 60 (34.88) | 54 (31.40) | 58 (33.72) |  |
| **Lipid Lowering Drug Use** |  |  |  |  | 0.700 |
| Yes | 484 (39.74) | 178 (36.78) | 143 (29.55) | 163 (33.68) |  |
| No | 734 (60.26) | 251 (34.20) | 225 (30.65) | 258 (35.15) |  |
| **Baseline Viral Load (copies/mL)** |  |  |  |  | 0.700 |
| < 200 | 393 (32.27) | 139 (35.37) | 126 (32.06) | 128 (32.57) |  |
| 200–10000 | 74 (6.08) | 27 (36.49) | 22 (29.73) | 25 (33.78) |  |
| ≥ 10000 | 63 (5.17) | 27 (42.86) | * | * |  |
| Unknown | 688 (56.49) | 236 (34.30) | * | * |  |
| **Baseline CD4 Count (cells/µL)** |  |  |  |  | 0.700 |
| < 200 | 56 (4.60) | * | * | * |  |
| 200–350 | 51 (4.19) | * | * | * |  |
| ≥ 350 | 320 (26.27) | 112 (35.00) | 100 (31.25) | 108 (33.75) |  |
| Unknown | 791 (64.94) | 276 (34.89) | 239 (30.21) | 276 (34.89) |  |
| **Baseline BMI (kg/m^2^)** |  |  |  |  | 0.400 |
| < 25 | 187 (15.35) | 61 (32.62) | 61 (32.62) | 65 (34.76) |  |
| 25–30 | 205 (16.83) | 70 (34.15) | 64 (31.22) | 71 (34.63) |  |
| ≥ 30 | 206 (16.91) | 71 (34.47) | 73 (35.44) | 62 (30.10) |  |
| Unknown | 620 (50.90) | 227 (36.61) | 170 (27.42) | 223 (35.97) |  |

^1^N (%).

^2^P values were calculated using Pearson’s Chi-square test for categorical variables and Welch’s two-sample t-test for continuous variables.

^3^Age groups 18–29 and 30–39 were combined into a single group 18–39 due to the extremely small size of the subpopulation.

^4^The “Other” sex group included participants who responded “Intersex”, “None”, “No matching concept”, “Skip”, and “Prefer not to answer”.

^5^The “Asian/Other/Unknown” race group refer to those who responded “Asian”, “More than one race”, “Other”, “Skip”, and “Prefer not to answer”.

*Counts less than 20 (and corresponding percentages) cannot be displayed due to NIH All of Us Research Program Data and Statistics Dissemination Policy. Some additional data were collapsed or obscured to prevent secondary calculation of these values.

**Supplementary Table 13. Factors associated with CVD in the sensitivity analysis using data balanced by PSM for the general PWH cohort**

|  | **AHR^1^ (95 % CI^2^)** | **p-value** |
| --- | --- | --- |
| **ART Regimen Type (Ref: Non-INSTI)** |  |  |
| Partial-INSTI | 0.59 (0.42, 0.82) | 0.001 |
| Only-INSTI | 0.34 (0.26, 0.46) | < 0.001 |
| **Age at Enrollment (Ref: 18–39^3^)** |  |  |
| 40–49 | 0.81 (0.38, 1.70) | 0.569 |
| 50–59 | 0.97 (0.52, 1.81) | 0.920 |
| 60–69 | 1.27 (0.68, 2.37) | 0.457 |
| ≥ 70 | 1.88 (0.96, 3.67) | 0.064 |
| **Sex (Ref: Female)** |  |  |
| Male | 0.77 (0.58, 1.02) | 0.069 |
| Other^4^ | 0.51 (0.24, 1.09) | 0.081 |
| **Race (Ref: White)** |  |  |
| Asian/Other/Unknown^5^ | 1.27 (0.63, 2.56) | 0.504 |
| Black or African American | 0.86 (0.63, 1.17) | 0.344 |
| **Ethnicity (Ref: Not Hispanic or Latino)** |  |  |
| Hispanic or Latino | 0.50 (0.25, 1.01) | 0.053 |
| Unknown | 0.54 (0.22, 1.32) | 0.173 |
| **Number of Historical Comorbidities (Ref: 0)** |  |  |
| 1 | 1.81 (1.34, 2.44) | < 0.001 |
| ≥ 2 | 3.43 (2.52, 4.67) | < 0.001 |
| **Lipid Lowering Drug Use (Ref: No)** |  |  |
| Yes | 1.35 (1.04, 1.75) | 0.023 |
| **Baseline Viral Load (copies/mL) (Ref: < 200)** |  |  |
| 200–10000 | 0.99 (0.58, 1.69) | 0.977 |
| ≥ 10000 | 2.32 (1.44, 3.74) | 0.001 |
| Unknown | 0.94 (0.70, 1.26) | 0.687 |
| **Baseline CD4 Count (cells/µL) (Ref: < 200)** |  |  |
| 200–350 | 1.32 (0.62, 2.79) | 0.475 |
| ≥ 350 | 1.03 (0.55, 1.90) | 0.935 |
| Unknown | 1.34 (0.74, 2.41) | 0.336 |
| **Baseline BMI (kg/m^2^) (Ref: < 25)** |  |  |
| 25–30 | 1.52 (0.95, 2.42) | 0.080 |
| ≥ 30 | 1.26 (0.79, 2.00) | 0.338 |
| Unknown | 0.80 (0.53, 1.20) | 0.272 |

^1^AHR: Adjusted hazard ratio; ^2^CI: Confidence interval.

^3^Age groups 18–29 and 30–39 were combined into a single group 18–39 due to the extremely small size of the subpopulation.

^4^The “Other” sex group included participants who responded “Intersex”, “None”, “No matching concept”, “Skip”, and “Prefer not to answer”.

^5^The “Asian/Other/Unknown” race group refer to those who responded “Asian”, “More than one race”, “Other”, “Skip”, and “Prefer not to answer”.

**Supplementary Table 14. Factors associated with CVD in the sensitivity analysis using data balanced by PSM for the INSTI-naïve cohort**

|  | **AHR^1^ (95 % CI^2^)** | **p-value** |
| --- | --- | --- |
| **Switch to INSTI-based Regimen (Ref: No)** |  |  |
| Yes | 0.29 (0.22, 0.40) | < 0.001 |
| **Age at Enrollment (Ref: 18–39^3^)** |  |  |
| 40–49 | 0.75 (0.27, 2.10) | 0.588 |
| 50–59 | 1.33 (0.57, 3.15) | 0.510 |
| 60–69 | 1.98 (0.84, 4.66) | 0.120 |
| ≥ 70 | 2.80 (1.15, 6.83) | 0.024 |
| **Sex (Ref: Female)** |  |  |
| Male | 0.81 (0.58, 1.13) | 0.215 |
| Other^4^ | 0.46 (0.18, 1.16) | 0.101 |
| **Race (Ref: White)** |  |  |
| Asian/Other/Unknown^5^ | 1.33 (0.56, 3.12) | 0.519 |
| Black or African American | 0.98 (0.68, 1.41) | 0.894 |
| **Ethnicity (Ref: Not Hispanic or Latino)** |  |  |
| Hispanic or Latino | 0.61 (0.26, 1.43) | 0.253 |
| Unknown | 0.62 (0.21, 1.85) | 0.395 |
| **Number of Historical Comorbidities (Ref: 0)** |  |  |
| 1 | 1.70 (1.21, 2.40) | 0.002 |
| ≥ 2 | 2.87 (1.96, 4.22) | < 0.001 |
| **Lipid Lowering Drug Use (Ref: No)** |  |  |
| Yes | 1.42 (1.05, 1.92) | 0.023 |
| **Baseline Viral Load (copies/mL) (Ref: < 200)** |  |  |
| 200–10000 | 0.87 (0.46, 1.65) | 0.674 |
| ≥ 10000 | 2.17 (1.21, 3.88) | 0.009 |
| Unknown | 1.03 (0.72, 1.48) | 0.865 |
| **Baseline CD4 Count (cells/µL) (Ref: < 200)** |  |  |
| 200–350 | 1.73 (0.57, 5.25) | 0.337 |
| ≥ 350 | 1.84 (0.70, 4.83) | 0.213 |
| Unknown | 2.41 (0.94, 6.19) | 0.066 |
| **Baseline BMI (kg/m^2^) (Ref: < 25)** |  |  |
| 25–30 | 1.36 (0.81, 2.28) | 0.250 |
| ≥ 30 | 0.95 (0.54, 1.69) | 0.871 |
| Unknown | 0.60 (0.38, 0.94) | 0.024 |

^1^AHR: Adjusted hazard ratio; ^2^CI: Confidence interval.

^3^Age groups 18–29 and 30–39 were combined into a single group 18–39 due to the extremely small size of the subpopulation.

^4^The “Other” sex group included participants who responded “Intersex”, “None”, “No matching concept”, “Skip”, and “Prefer not to answer”.

^5^The “Asian/Other/Unknown” race group refer to those who responded “Asian”, “More than one race”, “Other”, “Skip”, and “Prefer not to answer”.

**Supplementary Figure 1a. Log-log survival plot for assessing the proportional hazards (PH) assumption in the general PWH cohort**


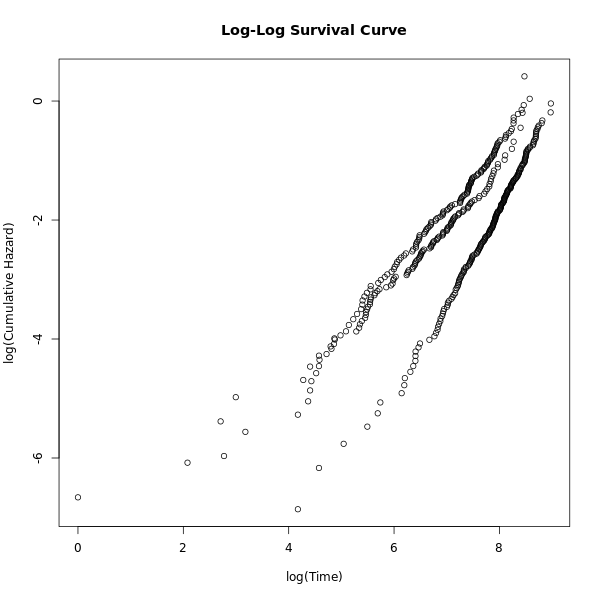


**Supplementary Figure 1b. Log-log survival plot for assessing the proportional hazards (PH) assumption in the INSTI-naïve cohort**


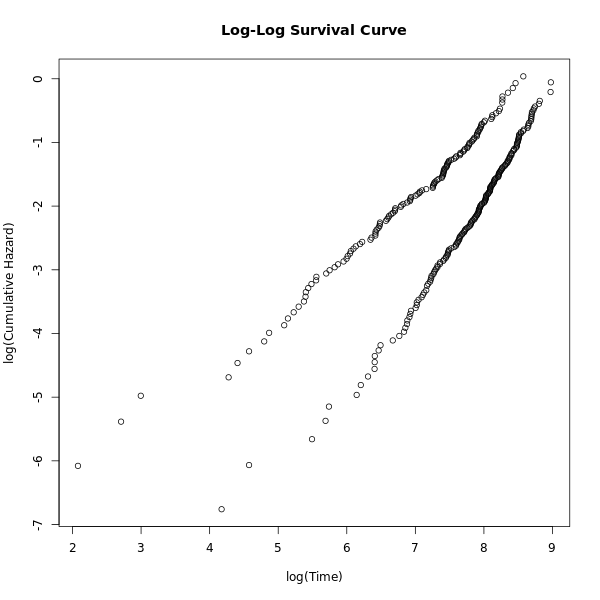

Supplement: 1 [file NIHMS2187110-supplement-1.docx]
