# Supplementary material for: Impact of integrase strand transfer inhibitors on cardiovascular disease in people with HIV
Source: Ann Epidemiol. Author manuscript; Available in PMC 2026 Jun 30. (PMC13318472; doi:10.1016/j.annepidem.2025.11.006)
Supplement: 2 [file NIHMS2187110-supplement-2.docx]

**Supplementary table 2. Antiretroviral therapy (ART) related RxNorm codes and corresponding medication labels used for data extraction.**

| RxNorm_ID | Preferred.Label |
| --- | --- |
| 2475415 | {1 (3 ML cabotegravir 200 MG/ML Injection) / 1 (3 ML rilpivirine 300 MG/ML Injection) } Pack |
| 1741731 | emtricitabine / rilpivirine / tenofovir alafenamide Pill |
| 1721612 | cobicistat / elvitegravir / emtricitabine / tenofovir alafenamide |
| 757694 | lamivudine 60 MG / nevirapine 100 MG / stavudine 12 MG Disintegrating Oral Tablet |
| 2055811 | doravirine / lamivudine / tenofovir disoproxil Oral Tablet [Delstrigo] |
| 205290 | efavirenz 200 MG Oral Capsule |
| 1802210 | dasabuvir / ombitasvir / paritaprevir / ritonavir Oral Product |
| 758555 | etravirine 100 MG Oral Tablet [Intelence] |
| 758553 | etravirine 100 MG [Intelence] |
| 1744007 | emtricitabine 133 MG / tenofovir disoproxil fumarate 200 MG Oral Tablet [Truvada] |
| 847749 | lopinavir 80 MG/ML / ritonavir 20 MG/ML Oral Solution [Kaletra] |
| 540398 | emtricitabine / tenofovir disoproxil Oral Tablet [Truvada] |
| 614534 | abacavir / lamivudine |
| 1857918 | maraviroc 20 MG/ML |
| 366448 | efavirenz Oral Capsule [Sustiva] |
| 1721611 | cobicistat / elvitegravir / emtricitabine / tenofovir alafenamide Oral Tablet |
| 1306295 | cobicistat / elvitegravir / emtricitabine / tenofovir disoproxil Oral Tablet [Stribild] |
| 565111 | lamivudine 150 MG [Epivir] |
| 900575 | ritonavir 100 MG Oral Tablet |
| 900624 | lamivudine / nevirapine / stavudine Oral Tablet |
| 1796081 | dolutegravir 25 MG Oral Tablet |
| 350652 | efavirenz 600 MG |
| 2055762 | doravirine 100 MG [Pifeltro] |
| 1235594 | raltegravir 25 MG [Isentress] |
| 2003246 | lamivudine / tenofovir disoproxil Oral Product |
| 364692 | ritonavir Oral Solution [Norvir] |
| 1433872 | dolutegravir Oral Tablet |
| 1989497 | dolutegravir / rilpivirine Pill |
| 757690 | lamivudine 30 MG / nevirapine 50 MG / stavudine 6 MG Disintegrating Oral Tablet |
| 1597388 | {2 (dasabuvir 250 MG Oral Tablet) / 2 (ombitasvir 12.5 MG / paritaprevir 75 MG / ritonavir 50 MG Oral Tablet) } Pack [Viekira Pak] |
| 1989506 | dolutegravir 50 MG / rilpivirine 25 MG Oral Tablet [Juluca] |
| 1597387 | {2 (dasabuvir 250 MG Oral Tablet) / 2 (ombitasvir 12.5 MG / paritaprevir 75 MG / ritonavir 50 MG Oral Tablet) } Pack |
| 1924312 | raltegravir 600 MG |
| 1747697 | emtricitabine 200 MG / tenofovir alafenamide 25 MG Oral Tablet [Descovy] |
| 2374566 | dolutegravir 5 MG Tablet for Oral Suspension [Tivicay] |
| 1741732 | emtricitabine / rilpivirine / tenofovir alafenamide Oral Tablet |
| 2475416 | {1 (3 ML cabotegravir 200 MG/ML Injection) / 1 (3 ML rilpivirine 300 MG/ML Injection) } Pack [CABENUVA 600 MG / 900 MG] |
| 316127 | lamivudine 150 MG |
| 1551993 | cobicistat 150 MG Oral Tablet |
| 372003 | efavirenz Oral Capsule |
| 744840 | raltegravir 400 MG |
| 2584352 | emtricitabine 120 MG |
| 728223 | maraviroc 150 MG Oral Tablet |
| 728221 | maraviroc 150 MG |
| 358299 | atazanavir sulfate |
| 1161887 | emtricitabine Oral Liquid Product |
| 329484 | abacavir 20 MG/ML |
| 1600703 | cobicistat / darunavir Oral Tablet |
| 573372 | lamivudine 5 MG/ML [Epivir HBV] |
| 729200 | maraviroc Oral Tablet [Selzentry] |
| 2380549 | 12 HR fostemsavir 600 MG Extended Release Oral Tablet |
| 757685 | lamivudine 150 MG / stavudine 30 MG Oral Tablet |
| 1102275 | rilpivirine 25 MG [Edurant] |
| 1163857 | lopinavir / ritonavir Oral Liquid Product |
| 900627 | lamivudine 150 MG / nevirapine 200 MG / stavudine 40 MG Oral Tablet |
| 573284 | efavirenz 200 MG [Sustiva] |
| 573286 | efavirenz 50 MG [Sustiva] |
| 1796080 | dolutegravir 25 MG |
| 744845 | raltegravir Oral Tablet [Isentress] |
| 1858267 | tenofovir alafenamide 25 MG Oral Tablet [Vemlidy] |
| 850456 | darunavir 150 MG [Prezista] |
| 1235593 | raltegravir 25 MG Chewable Tablet |
| 687428 | lamivudine / nevirapine / zidovudine |
| 372564 | lamivudine / zidovudine Oral Tablet |
| 372562 | lamivudine Oral Solution |
| 2584355 | bictegravir 30 MG / emtricitabine 120 MG / tenofovir alafenamide 15 MG [Biktarvy] |
| 729203 | maraviroc 300 MG Oral Tablet [Selzentry] |
| 1162812 | lamivudine / nevirapine / zidovudine Oral Product |
| 2122526 | dolutegravir 50 MG / lamivudine 300 MG Oral Tablet [Dovato] |
| 1162811 | lamivudine / nevirapine / stavudine Pill |
| 847743 | lopinavir 100 MG / ritonavir 25 MG [Kaletra] |
| 1147336 | emtricitabine 200 MG / rilpivirine 25 MG / tenofovir disoproxil fumarate 300 MG [Complera] |
| 1924313 | raltegravir 600 MG Oral Tablet |
| 1306290 | cobicistat / elvitegravir / emtricitabine / tenofovir disoproxil Oral Tablet |
| 317359 | efavirenz 200 MG |
| 2380537 | fostemsavir Pill |
| 213461 | abacavir 20 MG/ML Oral Solution [Ziagen] |
| 1989498 | dolutegravir / rilpivirine Oral Tablet |
| 1145800 | emtricitabine / rilpivirine / tenofovir disoproxil Oral Tablet |
| 597291 | emtricitabine 10 MG/ML Oral Solution |
| 1433873 | dolutegravir 50 MG Oral Tablet |
| 1924314 | raltegravir 600 MG [Isentress] |
| 1999660 | bictegravir |
| 402094 | atazanavir 150 MG Oral Capsule [Reyataz] |
| 1744008 | emtricitabine 167 MG |
| 1551995 | cobicistat 150 MG [Tybost] |
| 754759 | etravirine 100 MG |
| 850454 | darunavir 150 MG |
| 602395 | abacavir 600 MG / lamivudine 300 MG Oral Tablet [Epzicom] |
| 1999673 | bictegravir 50 MG / emtricitabine 200 MG / tenofovir alafenamide 25 MG Oral Tablet [Biktarvy] |
| 2584353 | tenofovir alafenamide 15 MG |
| 1433879 | dolutegravir 50 MG Oral Tablet [Tivicay] |
| 1857919 | maraviroc Oral Liquid Product |
| 1162810 | lamivudine / nevirapine / stavudine Oral Product |
| 1546890 | abacavir 600 MG / dolutegravir 50 MG / lamivudine 300 MG [Triumeq] |
| 460132 | darunavir |
| 1551980 | elvitegravir Oral Product |
| 1161888 | emtricitabine Oral Product |
| 366398 | ritonavir Oral Capsule [Norvir] |
| 1007761 | efavirenz / emtricitabine / tenofovir disoproxil |
| 1147334 | emtricitabine 200 MG / rilpivirine 25 MG / tenofovir disoproxil fumarate 300 MG Oral Tablet |
| 2001423 | efavirenz 400 MG |
| 1926066 | ritonavir 100 MG Oral Powder |
| 284620 | abacavir / lamivudine / zidovudine |
| 1163859 | lopinavir / ritonavir Pill |
| 1165486 | ritonavir Oral Product |
| 1235595 | raltegravir 25 MG Chewable Tablet [Isentress] |
| 794609 | darunavir 600 MG [Prezista] |
| 687064 | lamivudine 150 MG / nevirapine 200 MG / zidovudine 300 MG Oral Tablet |
| 2003247 | lamivudine / tenofovir disoproxil Pill |
| 2043313 | ibalizumab-uiyk |
| 1857921 | maraviroc 20 MG/ML Oral Solution |
| 1102277 | rilpivirine 25 MG Oral Tablet [Edurant] |
| 2475417 | {1 (2 ML cabotegravir 200 MG/ML Injection) / 1 (2 ML rilpivirine 300 MG/ML Injection) } Pack |
| 1421185 | raltegravir Chewable Product |
| 1162813 | lamivudine / nevirapine / zidovudine Pill |
| 1741733 | emtricitabine 200 MG / rilpivirine 25 MG / tenofovir alafenamide 25 MG Oral Tablet |
| 576102 | atazanavir 150 MG [Reyataz] |
| 2475201 | cabotegravir 30 MG [Vocabria] |
| 350655 | lamivudine 300 MG |
| 1924315 | raltegravir 600 MG Oral Tablet [Isentress] |
| 643068 | efavirenz 600 MG / emtricitabine 200 MG / tenofovir disoproxil fumarate 300 MG [Atripla] |
| 1306291 | cobicistat / elvitegravir / emtricitabine / tenofovir disoproxil |
| 332450 | ritonavir 20 MG/ML |
| 1250720 | etravirine 25 MG |
| 2055813 | doravirine 100 MG / lamivudine 300 MG / tenofovir disoproxil fumarate 300 MG Oral Tablet |
| 2475195 | cabotegravir 30 MG |
| 1145801 | emtricitabine / rilpivirine / tenofovir disoproxil |
| 2123030 | lamivudine 300 MG / tenofovir disoproxil fumarate 300 MG [Temixys] |
| 1598388 | lamivudine / tenofovir disoproxil |
| 213088 | lamivudine 150 MG / zidovudine 300 MG Oral Tablet [Combivir] |
| 1721619 | cobicistat 150 MG / elvitegravir 150 MG / emtricitabine 200 MG / tenofovir alafenamide 10 MG Oral Tablet [Genvoya] |
| 1741739 | emtricitabine 200 MG / rilpivirine 25 MG / tenofovir alafenamide 25 MG Oral Tablet [Odefsey] |
| 1857920 | maraviroc Oral Solution |
| 643072 | darunavir 300 MG |
| 2049668 | cobicistat / darunavir / emtricitabine / tenofovir alafenamide Oral Product |
| 1744009 | emtricitabine 167 MG / tenofovir disoproxil fumarate 250 MG Oral Tablet |
| 1102276 | rilpivirine Oral Tablet [Edurant] |
| 343047 | atazanavir |
| 1163858 | lopinavir / ritonavir Oral Product |
| 1926065 | ritonavir Oral Powder |
| 2380551 | 12 HR fostemsavir 600 MG Extended Release Oral Tablet [Rukobia] |
| 597730 | lopinavir 200 MG / ritonavir 50 MG Oral Tablet |
| 402423 | efavirenz Oral Solution |
| 1163784 | etravirine Pill |
| 1486841 | raltegravir 100 MG Granules for Oral Suspension [Isentress] |
| 1147337 | emtricitabine 200 MG / rilpivirine 25 MG / tenofovir disoproxil fumarate 300 MG Oral Tablet [Complera] |
| 900630 | lamivudine 30 MG / zidovudine 60 MG Oral Tablet |
| 1306298 | cobicistat 150 MG / elvitegravir 150 MG / emtricitabine 200 MG / tenofovir disoproxil fumarate 300 MG Oral Tablet [Stribild] |
| 616146 | emtricitabine 10 MG/ML [Emtriva] |
| 1598982 | atazanavir 50 MG |
| 1601657 | atazanavir / cobicistat Oral Tablet [Evotaz] |
| 2584356 | bictegravir 30 MG / emtricitabine 120 MG / tenofovir alafenamide 15 MG Oral Tablet [Biktarvy] |
| 847739 | lopinavir 200 MG / ritonavir 50 MG [Kaletra] |
| 2049671 | cobicistat 150 MG / darunavir 800 MG / emtricitabine 200 MG / tenofovir alafenamide 10 MG Oral Tablet |
| 2049670 | cobicistat / darunavir / emtricitabine / tenofovir alafenamide Oral Tablet |
| 1999661 | bictegravir sodium |
| 2055756 | doravirine 100 MG |
| 1165487 | ritonavir Pill |
| 364697 | lamivudine Oral Solution [Epivir] |
| 402335 | atazanavir 100 MG |
| 643077 | darunavir Oral Tablet [Prezista] |
| 373796 | ritonavir Oral Solution |
| 1601650 | atazanavir / cobicistat Oral Product |
| 2003249 | lamivudine 300 MG / tenofovir disoproxil fumarate 300 MG Oral Tablet |
| 1433875 | dolutegravir 50 MG [Tivicay] |
| 349477 | efavirenz 600 MG Oral Tablet |
| 2003248 | lamivudine / tenofovir disoproxil Oral Tablet |
| 2380538 | fostemsavir Extended Release Oral Tablet |
| 1008145 | emtricitabine / tenofovir disoproxil |
| 205291 | efavirenz 100 MG Oral Capsule |
| 1989499 | dolutegravir / rilpivirine |
| 1157184 | darunavir Oral Product |
| 1551996 | cobicistat Oral Tablet [Tybost] |
| 847748 | lopinavir / ritonavir Oral Solution [Kaletra] |
| 1235590 | raltegravir Chewable Tablet [Isentress] |
| 1597375 | ombitasvir / paritaprevir / ritonavir Oral Product |
| 2590641 | emtricitabine 120 MG / tenofovir alafenamide 15 MG Oral Tablet |
| 2475418 | {1 (2 ML cabotegravir 200 MG/ML Injection) / 1 (2 ML rilpivirine 300 MG/ML Injection) } Pack [CABENUVA 400 MG / 600 MG] |
| 402247 | atazanavir 200 MG Oral Capsule |
| 406016 | emtricitabine 200 MG |
| 1601656 | atazanavir 300 MG / cobicistat 150 MG [Evotaz] |
| 2001424 | efavirenz 400 MG / lamivudine 300 MG / tenofovir disoproxil fumarate 300 MG Oral Tablet |
| 900574 | ritonavir Oral Tablet |
| 1744011 | emtricitabine 167 MG / tenofovir disoproxil fumarate 250 MG Oral Tablet [Truvada] |
| 2584354 | bictegravir 30 MG / emtricitabine 120 MG / tenofovir alafenamide 15 MG Oral Tablet |
| 639887 | emtricitabine 200 MG / tenofovir disoproxil fumarate 300 MG [Truvada] |
| 368309 | lamivudine / zidovudine Oral Tablet [Combivir] |
| 1546891 | abacavir / dolutegravir / lamivudine Oral Tablet [Triumeq] |
| 1744010 | emtricitabine 167 MG / tenofovir disoproxil fumarate 250 MG [Truvada] |
| 1600259 | dasabuvir / ombitasvir / paritaprevir / ritonavir |
| 1161889 | emtricitabine Pill |
| 1163783 | etravirine Oral Product |
| 402370 | atazanavir Oral Capsule |
| 1250721 | etravirine 25 MG Oral Tablet |
| 315853 | efavirenz 50 MG |
| 1926067 | ritonavir Oral Powder [Norvir] |
| 1052660 | etravirine 200 MG Oral Tablet [Intelence] |
| 1161284 | efavirenz / lamivudine / tenofovir disoproxil Oral Product |
| 729199 | maraviroc 150 MG [Selzentry] |
| 2043314 | ibalizumab-uiyk 150 MG/ML |
| 746645 | lopinavir 100 MG / ritonavir 25 MG Oral Tablet |
| 1857922 | maraviroc 20 MG/ML [Selzentry] |
| 476143 | abacavir / lamivudine Oral Tablet |
| 2475202 | cabotegravir Oral Tablet [Vocabria] |
| 575077 | abacavir 300 MG / lamivudine 150 MG / zidovudine 300 MG [Trizivir] |
| 1165075 | maraviroc Oral Product |
| 1598983 | atazanavir Oral Powder Product |
| 1165076 | maraviroc Pill |
| 1162814 | lamivudine / stavudine Oral Product |
| 2594978 | 2 ML cabotegravir 200 MG/ML Injection |
| 2055814 | doravirine 100 MG / lamivudine 300 MG / tenofovir disoproxil fumarate 300 MG [Delstrigo] |
| 2123031 | lamivudine / tenofovir disoproxil Oral Tablet [Temixys] |
| 2475196 | cabotegravir Oral Product |
| 2055758 | doravirine Pill |
| 2055757 | doravirine Oral Product |
| 831868 | darunavir 75 MG Oral Tablet |
| 1601652 | atazanavir / cobicistat Oral Tablet |
| 1306292 | cobicistat 150 MG / elvitegravir 150 MG / emtricitabine 200 MG / tenofovir disoproxil fumarate 300 MG Oral Tablet |
| 1601651 | atazanavir / cobicistat Pill |
| 744844 | raltegravir 400 MG [Isentress] |
| 757686 | lamivudine 30 MG |
| 2049669 | cobicistat / darunavir / emtricitabine / tenofovir alafenamide Pill |
| 575950 | lamivudine 300 MG [Epivir] |
| 1235591 | raltegravir 100 MG Chewable Tablet [Isentress] |
| 2590642 | emtricitabine 120 MG / tenofovir alafenamide 15 MG [Descovy] |
| 1597376 | ombitasvir / paritaprevir / ritonavir Pill |
| 900577 | ritonavir 100 MG Oral Tablet [Norvir] |
| 1250723 | etravirine 25 MG Oral Tablet [Intelence] |
| 2598346 | abacavir / dolutegravir / lamivudine Oral Liquid Product |
| 372563 | lamivudine Oral Tablet |
| 1721603 | tenofovir alafenamide |
| 2598345 | abacavir 60 MG |
| 847740 | lopinavir / ritonavir Oral Tablet [Kaletra] |
| 2122516 | dolutegravir / lamivudine Pill |
| 317397 | lamivudine 100 MG |
| 68244 | lamivudine |
| 539824 | abacavir / lamivudine Oral Tablet [Epzicom] |
| 1857923 | maraviroc Oral Solution [Selzentry] |
| 1163058 | abacavir / lamivudine / zidovudine Oral Product |
| 1796076 | dolutegravir 10 MG |
| 573371 | lamivudine 100 MG [Epivir HBV] |
| 1161285 | efavirenz / lamivudine / tenofovir disoproxil Pill |
| 597290 | emtricitabine Oral Solution |
| 200082 | lamivudine 150 MG / zidovudine 300 MG Oral Tablet |
| 1162815 | lamivudine / stavudine Pill |
| 2594979 | rilpivirine Injection |
| 643066 | efavirenz 600 MG / emtricitabine 200 MG / tenofovir disoproxil fumarate 300 MG Oral Tablet |
| 1157185 | darunavir Pill |
| 307650 | abacavir 300 MG / lamivudine 150 MG / zidovudine 300 MG Oral Tablet |
| 1721608 | tenofovir alafenamide 10 MG |
| 2003251 | lamivudine 300 MG / tenofovir disoproxil fumarate 300 MG [Cimduo] |
| 1598984 | atazanavir Oral Powder |
| 370502 | abacavir Oral Solution |
| 152931 | lamivudine 150 MG Oral Tablet [Epivir] |
| 1727491 | emtricitabine / tenofovir alafenamide |
| 1250722 | etravirine 25 MG [Intelence] |
| 602392 | abacavir 600 MG |
| 602394 | abacavir 600 MG / lamivudine 300 MG [Epzicom] |
| 2055759 | doravirine Oral Tablet |
| 2475197 | cabotegravir Pill |
| 1999664 | bictegravir / emtricitabine / tenofovir alafenamide Oral Product |
| 2055815 | doravirine 100 MG / lamivudine 300 MG / tenofovir disoproxil fumarate 300 MG Oral Tablet [Delstrigo] |
| 316647 | ritonavir 80 MG/ML |
| 2122515 | dolutegravir / lamivudine Oral Product |
| 1601653 | atazanavir / cobicistat |
| 2380373 | fostemsavir |
| 1597379 | ombitasvir 12.5 MG / paritaprevir 75 MG / ritonavir 50 MG Oral Tablet |
| 1154365 | atazanavir Pill |
| 221052 | abacavir sulfate |
| 757689 | lamivudine / nevirapine / stavudine Disintegrating Oral Tablet |
| 317488 | ritonavir 100 MG |
| 1858259 | tenofovir alafenamide Pill |
| 1236626 | darunavir Oral Liquid Product |
| 1926064 | ritonavir Oral Powder Product |
| 1551999 | cobicistat 150 MG Oral Tablet [Tybost] |
| 2049673 | cobicistat 150 MG / darunavir 800 MG / emtricitabine 200 MG / tenofovir alafenamide 10 MG [Symtuza] |
| 311369 | lopinavir 80 MG/ML / ritonavir 20 MG/ML Oral Solution |
| 1163060 | abacavir / lamivudine Oral Product |
| 1546894 | abacavir 600 MG / dolutegravir 50 MG / lamivudine 300 MG Oral Tablet [Triumeq] |
| 2598347 | abacavir / dolutegravir / lamivudine Tablet for Oral Suspension |
| 1855366 | raltegravir Granule Product |
| 2588045 | cabotegravir 200 MG/ML [Apretude] |
| 2475407 | cabotegravir 200 MG/ML |
| 794610 | darunavir 600 MG Oral Tablet [Prezista] |
| 576513 | emtricitabine 200 MG [Emtriva] |
| 1857925 | maraviroc 20 MG/ML Oral Solution [Selzentry] |
| 2594980 | 3 ML rilpivirine 300 MG/ML Injection |
| 199148 | lamivudine 10 MG/ML Oral Solution |
| 1999663 | bictegravir 50 MG |
| 754760 | etravirine Oral Tablet |
| 1165469 | rilpivirine Oral Product |
| 1433869 | dolutegravir 50 MG |
| 364596 | abacavir Oral Solution [Ziagen] |
| 1597377 | ombitasvir / paritaprevir / ritonavir Oral Tablet |
| 1235592 | raltegravir 25 MG |
| 2590643 | emtricitabine 120 MG / tenofovir alafenamide 15 MG Oral Tablet [Descovy] |
| 1858258 | tenofovir alafenamide Oral Product |
| 1236625 | darunavir 100 MG/ML |
| 1088481 | efavirenz 200 MG Oral Tablet |
| 1741729 | tenofovir alafenamide 25 MG |
| 1857910 | maraviroc 25 MG |
| 1154364 | atazanavir Oral Product |
| 1597378 | ombitasvir / paritaprevir / ritonavir |
| 576101 | atazanavir 200 MG [Reyataz] |
| 2122517 | dolutegravir / lamivudine Oral Tablet |
| 402422 | efavirenz 30 MG/ML |
| 2475199 | cabotegravir 30 MG Oral Tablet |
| 1999666 | bictegravir / emtricitabine / tenofovir alafenamide Oral Tablet |
| 1858260 | tenofovir alafenamide Oral Tablet |
| 1306288 | cobicistat / elvitegravir / emtricitabine / tenofovir disoproxil Oral Product |
| 1721604 | tenofovir alafenamide fumarate |
| 1145805 | emtricitabine / rilpivirine / tenofovir disoproxil Oral Tablet [Complera] |
| 2123034 | lamivudine 300 MG / tenofovir disoproxil fumarate 300 MG Oral Tablet [Temixys] |
| 85762 | ritonavir |
| 2122518 | dolutegravir / lamivudine |
| 1161286 | efavirenz / emtricitabine / tenofovir disoproxil Oral Product |
| 1600710 | cobicistat 150 MG / darunavir 800 MG Oral Tablet [Prezcobix] |
| 2380536 | fostemsavir Oral Product |
| 1796077 | dolutegravir 10 MG Oral Tablet |
| 402334 | atazanavir 150 MG |
| 402332 | atazanavir 200 MG |
| 1163059 | abacavir / lamivudine / zidovudine Pill |
| 373795 | ritonavir Oral Capsule |
| 1926069 | ritonavir 100 MG Oral Powder [Norvir] |
| 1855367 | raltegravir Granules for Oral Suspension |
| 402246 | atazanavir 150 MG Oral Capsule |
| 2003252 | lamivudine / tenofovir disoproxil Oral Tablet [Cimduo] |
| 2475205 | cabotegravir 30 MG Oral Tablet [Vocabria] |
| 476556 | emtricitabine 200 MG / tenofovir disoproxil fumarate 300 MG Oral Tablet |
| 1162816 | lamivudine / zidovudine Oral Product |
| 1721609 | cobicistat / elvitegravir / emtricitabine / tenofovir alafenamide Oral Product |
| 284640 | lopinavir / ritonavir |
| 719872 | raltegravir |
| 152971 | ritonavir 80 MG/ML Oral Solution [Norvir] |
| 1546888 | abacavir 600 MG / dolutegravir 50 MG / lamivudine 300 MG Oral Tablet |
| 1165470 | rilpivirine Pill |
| 1598986 | atazanavir 50 MG [Reyataz] |
| 847747 | lopinavir 80 MG/ML / ritonavir 20 MG/ML [Kaletra] |
| 1598985 | atazanavir 50 MG Oral Powder |
| 317150 | ritonavir 100 MG Oral Capsule |
| 2380374 | fostemsavir tromethamine |
| 195085 | efavirenz |
| 1601654 | atazanavir 300 MG / cobicistat 150 MG Oral Tablet |
| 1727492 | emtricitabine / rilpivirine / tenofovir alafenamide |
| 728225 | maraviroc 300 MG Oral Tablet |
| 1306287 | elvitegravir 150 MG |
| 368247 | lamivudine Oral Tablet [Epivir HBV] |
| 1999665 | bictegravir / emtricitabine / tenofovir alafenamide Pill |
| 2475198 | cabotegravir Oral Tablet |
| 367794 | lamivudine Oral Tablet [Epivir] |
| 729202 | maraviroc 300 MG [Selzentry] |
| 1161303 | emtricitabine / rilpivirine / tenofovir disoproxil Oral Product |
| 1857912 | maraviroc 25 MG [Selzentry] |
| 2599543 | {10 (nirmatrelvir 150 MG Oral Tablet) / 10 (ritonavir 100 MG Oral Tablet) } Pack [Paxlovid 150 MG /100 MG Dose Pack] |
| 643074 | darunavir 300 MG Oral Tablet |
| 1236627 | darunavir Oral Suspension |
| 1235586 | raltegravir 100 MG |
| 213484 | lamivudine 5 MG/ML Oral Solution [Epivir HBV] |
| 864660 | efavirenz / lamivudine / tenofovir disoproxil Oral Tablet |
| 2122519 | dolutegravir 50 MG / lamivudine 300 MG Oral Tablet |
| 2475408 | cabotegravir Injectable Product |
| 2049674 | cobicistat / darunavir / emtricitabine / tenofovir alafenamide Oral Tablet [Symtuza] |
| 1163061 | abacavir / lamivudine Pill |
| 1747688 | emtricitabine / tenofovir alafenamide Oral Product |
| 1747690 | emtricitabine / tenofovir alafenamide Oral Tablet |
| 597289 | emtricitabine 10 MG/ML |
| 847745 | lopinavir 100 MG / ritonavir 25 MG Oral Tablet [Kaletra] |
| 2598348 | abacavir 60 MG / dolutegravir 5 MG / lamivudine 30 MG Tablet for Oral Suspension |
| 2055755 | doravirine |
| 404587 | emtricitabine 200 MG Oral Capsule [Emtriva] |
| 1161287 | efavirenz / emtricitabine / tenofovir disoproxil Pill |
| 2043317 | 1.33 ML ibalizumab-uiyk 150 MG/ML Injection |
| 2588077 | 3 ML cabotegravir 200 MG/ML Injection |
| 1744001 | emtricitabine 100 MG / tenofovir disoproxil fumarate 150 MG Oral Tablet |
| 831867 | darunavir 75 MG |
| 831869 | darunavir 75 MG [Prezista] |
| 2588076 | cabotegravir Injection |
| 1796078 | dolutegravir 10 MG [Tivicay] |
| 1600704 | cobicistat 150 MG / darunavir 800 MG Oral Tablet |
| 1744000 | emtricitabine 100 MG |
| 1857911 | maraviroc 25 MG Oral Tablet |
| 2599542 | {10 (nirmatrelvir 150 MG Oral Tablet) / 10 (ritonavir 100 MG Oral Tablet) } Pack |
| 331537 | ritonavir 33.3 MG |
| 824338 | darunavir 400 MG Oral Tablet |
| 2374559 | dolutegravir 5 MG |
| 573349 | abacavir 20 MG/ML [Ziagen] |
| 1162817 | lamivudine / zidovudine Pill |
| 687063 | lamivudine / nevirapine / zidovudine Oral Tablet |
| 1598987 | atazanavir Oral Powder [Reyataz] |
| 2380550 | fostemsavir 600 MG [Rukobia] |
| 847741 | lopinavir 200 MG / ritonavir 50 MG Oral Tablet [Kaletra] |
| 406018 | emtricitabine Oral Capsule [Emtriva] |
| 1102270 | rilpivirine |
| 565112 | lamivudine 10 MG/ML [Epivir] |
| 597729 | lopinavir / ritonavir Oral Tablet |
| 1999667 | bictegravir 50 MG / emtricitabine 200 MG / tenofovir alafenamide 25 MG Oral Tablet |
| 1858261 | tenofovir alafenamide 25 MG Oral Tablet |
| 1306289 | cobicistat / elvitegravir / emtricitabine / tenofovir disoproxil Pill |
| 1235587 | raltegravir Chewable Tablet |
| 1236628 | darunavir 100 MG/ML Oral Suspension |
| 1857914 | maraviroc 75 MG |
| 1165485 | ritonavir Oral Liquid Product |
| 368582 | efavirenz Oral Tablet [Sustiva] |
| 1236629 | darunavir 100 MG/ML [Prezista] |
| 670026 | darunavir 600 MG Oral Tablet |
| 1855369 | raltegravir Granules for Oral Suspension [Isentress] |
| 2598349 | abacavir 60 MG / dolutegravir 5 MG / lamivudine 30 MG [Triumeq] |
| 368153 | abacavir / lamivudine / zidovudine Oral Tablet [Trizivir] |
| 729583 | lamivudine 150 MG / stavudine 40 MG Oral Tablet |
| 370503 | abacavir Oral Tablet |
| 758660 | lamivudine / nevirapine / stavudine |
| 744841 | raltegravir Oral Tablet |
| 402008 | efavirenz 30 MG/ML Oral Solution |
| 1747689 | emtricitabine / tenofovir alafenamide Pill |
| 643065 | efavirenz / emtricitabine / tenofovir disoproxil Oral Tablet |
| 1163062 | abacavir Oral Liquid Product |
| 1747691 | emtricitabine 200 MG / tenofovir alafenamide 25 MG Oral Tablet |
| 1306284 | cobicistat |
| 352143 | efavirenz 600 MG Oral Tablet [Sustiva] |
| 620216 | maraviroc |
| 374643 | lopinavir / ritonavir Oral Solution |
| 2374560 | dolutegravir Oral Liquid Product |
| 2594982 | 2 ML rilpivirine 300 MG/ML Injection |
| 1052657 | etravirine 200 MG |
| 1598380 | cobicistat / darunavir |
| 1744002 | emtricitabine 100 MG / tenofovir disoproxil fumarate 150 MG [Truvada] |
| 2055763 | doravirine Oral Tablet [Pifeltro] |
| 1162819 | lamivudine Oral Product |
| 2588080 | 3 ML cabotegravir 200 MG/ML Injection [Apretude] |
| 1161304 | emtricitabine / rilpivirine / tenofovir disoproxil Pill |
| 1598989 | atazanavir 50 MG Oral Powder [Reyataz] |
| 2101004 | efavirenz 400 MG / lamivudine 300 MG / tenofovir disoproxil fumarate 300 MG [Symfi] |
| 1857913 | maraviroc 25 MG Oral Tablet [Selzentry] |
| 2475077 | cabotegravir |
| 1359268 | darunavir 800 MG |
| 2003255 | lamivudine 300 MG / tenofovir disoproxil fumarate 300 MG Oral Tablet [Cimduo] |
| 729581 | lamivudine / stavudine |
| 1102271 | rilpivirine 25 MG |
| 1359270 | darunavir 800 MG [Prezista] |
| 664741 | atazanavir 300 MG Oral Capsule |
| 664743 | atazanavir 300 MG Oral Capsule [Reyataz] |
| 1989500 | dolutegravir 50 MG / rilpivirine 25 MG Oral Tablet |
| 1546884 | abacavir / dolutegravir / lamivudine Oral Product |
| 616148 | emtricitabine 10 MG/ML Oral Solution [Emtriva] |
| 475969 | etravirine |
| 368253 | abacavir Oral Tablet [Ziagen] |
| 1858263 | tenofovir alafenamide 25 MG [Vemlidy] |
| 1236630 | darunavir Oral Suspension [Prezista] |
| 1796079 | dolutegravir 10 MG Oral Tablet [Tivicay] |
| 1996260 | cobicistat / darunavir / emtricitabine / tenofovir alafenamide |
| 2001430 | efavirenz 400 MG / lamivudine 300 MG / tenofovir disoproxil fumarate 300 MG Oral Tablet [Symfi] |
| 831870 | darunavir 75 MG Oral Tablet [Prezista] |
| 152932 | lamivudine 10 MG/ML Oral Solution [Epivir] |
| 1433867 | dolutegravir sodium |
| 1161288 | efavirenz Oral Liquid Product |
| 565135 | ritonavir 80 MG/ML [Norvir] |
| 602393 | abacavir 600 MG / lamivudine 300 MG Oral Tablet |
| 2055805 | doravirine / lamivudine / tenofovir disoproxil Oral Product |
| 754761 | etravirine 100 MG Oral Tablet |
| 199147 | lamivudine 150 MG Oral Tablet |
| 205328 | lamivudine 100 MG Oral Tablet |
| 746644 | ritonavir 25 MG |
| 2475076 | cabotegravir sodium |
| 315852 | efavirenz 100 MG |
| 1295256 | lamivudine / nevirapine / stavudine Disintegrating Oral Product |
| 1163063 | abacavir Oral Product |
| 1162818 | lamivudine Oral Liquid Product |
| 1052659 | etravirine 200 MG [Intelence] |
| 242680 | abacavir 20 MG/ML Oral Solution |
| 1162820 | lamivudine Pill |
| 213392 | efavirenz 50 MG Oral Capsule [Sustiva] |
| 213390 | efavirenz 200 MG Oral Capsule [Sustiva] |
| 1744004 | emtricitabine 133 MG |
| 311368 | lopinavir 133 MG / ritonavir 33.3 MG Oral Capsule |
| 1306286 | elvitegravir |
| 403875 | emtricitabine 200 MG Oral Capsule |
| 2374562 | dolutegravir 5 MG Tablet for Oral Suspension |
| 644682 | darunavir ethanolate |
| 364593 | lamivudine Oral Solution [Epivir HBV] |
| 199249 | ritonavir 80 MG/ML Oral Solution |
| 1235588 | raltegravir 100 MG Chewable Tablet |
| 575878 | efavirenz 600 MG [Sustiva] |
| 1857915 | maraviroc 75 MG Oral Tablet |
| 1359269 | darunavir 800 MG Oral Tablet |
| 643070 | efavirenz 600 MG / emtricitabine 200 MG / tenofovir disoproxil fumarate 300 MG Oral Tablet [Atripla] |
| 285028 | abacavir 300 MG / lamivudine 150 MG / zidovudine 300 MG Oral Tablet [Trizivir] |
| 1996259 | bictegravir / emtricitabine / tenofovir alafenamide |
| 758554 | etravirine Oral Tablet [Intelence] |
| 2043460 | efavirenz 600 MG / lamivudine 300 MG / tenofovir disoproxil fumarate 300 MG [Symfi] |
| 1486838 | raltegravir 100 MG Granules for Oral Suspension |
| 2588079 | cabotegravir Injection [Apretude] |
| 378299 | efavirenz Oral Tablet |
| 1744003 | emtricitabine 100 MG / tenofovir disoproxil fumarate 150 MG Oral Tablet [Truvada] |
| 276237 | emtricitabine |
| 2598351 | abacavir / dolutegravir / lamivudine Tablet for Oral Suspension [Triumeq] |
| 729201 | maraviroc 150 MG Oral Tablet [Selzentry] |
| 1306285 | cobicistat 150 MG |
| 1600706 | cobicistat 150 MG / darunavir 800 MG [Prezcobix] |
| 900625 | lamivudine 150 MG / nevirapine 200 MG / stavudine 30 MG Oral Tablet |
| 2374561 | dolutegravir Tablet for Oral Suspension |
| 1433868 | dolutegravir |
| 1161289 | efavirenz Oral Product |
| 900576 | ritonavir Oral Tablet [Norvir] |
| 728224 | maraviroc 300 MG |
| 728222 | maraviroc Oral Tablet |
| 2043319 | ibalizumab-uiyk 150 MG/ML [Trogarzo] |
| 1052658 | etravirine 200 MG Oral Tablet |
| 2055806 | doravirine / lamivudine / tenofovir disoproxil Pill |
| 1721613 | cobicistat 150 MG / elvitegravir 150 MG / emtricitabine 200 MG / tenofovir alafenamide 10 MG Oral Tablet |
| 643073 | darunavir Oral Tablet |
| 402333 | atazanavir Oral Capsule [Reyataz] |
| 757691 | lamivudine 60 MG |
| 2380542 | fostemsavir Extended Release Oral Tablet [Rukobia] |
| 1796083 | dolutegravir 25 MG Oral Tablet [Tivicay] |
| 213483 | lamivudine 100 MG Oral Tablet [Epivir HBV] |
| 242692 | lamivudine 5 MG/ML Oral Solution |
| 190521 | abacavir |
| 152970 | ritonavir 100 MG Oral Capsule [Norvir] |
| 1858264 | tenofovir alafenamide Oral Tablet [Vemlidy] |
| 316128 | lamivudine 5 MG/ML |
| 316126 | lamivudine 10 MG/ML |
| 402245 | atazanavir 100 MG Oral Capsule |
| 1999669 | bictegravir 50 MG / emtricitabine 200 MG / tenofovir alafenamide 25 MG [Biktarvy] |
| 864661 | efavirenz 600 MG / lamivudine 300 MG / tenofovir disoproxil fumarate 300 MG Oral Tablet |
| 1102272 | rilpivirine Oral Tablet |
| 1359271 | darunavir 800 MG Oral Tablet [Prezista] |
| 540395 | emtricitabine / tenofovir disoproxil Oral Tablet |
| 1546885 | abacavir / dolutegravir / lamivudine Pill |
| 1008789 | efavirenz / lamivudine / tenofovir disoproxil |
| 329485 | abacavir 300 MG |
| 573017 | lamivudine 150 MG / zidovudine 300 MG [Combivir] |
| 2122522 | dolutegravir 50 MG / lamivudine 300 MG [Dovato] |
| 2475411 | rilpivirine 300 MG/ML |
| 349491 | lamivudine 300 MG Oral Tablet |
| 1235589 | raltegravir 100 MG [Isentress] |
| 1857916 | maraviroc 75 MG [Selzentry] |
| 1796082 | dolutegravir 25 MG [Tivicay] |
| 2043461 | efavirenz / lamivudine / tenofovir disoproxil Oral Tablet [Symfi] |
| 2049677 | cobicistat 150 MG / darunavir 800 MG / emtricitabine 200 MG / tenofovir alafenamide 10 MG Oral Tablet [Symtuza] |
| 374813 | abacavir / lamivudine / zidovudine Oral Tablet |
| 1163064 | abacavir Pill |
| 1747693 | emtricitabine 200 MG / tenofovir alafenamide 25 MG [Descovy] |
| 744846 | raltegravir 400 MG Oral Tablet [Isentress] |
| 850457 | darunavir 150 MG Oral Tablet [Prezista] |
| 850455 | darunavir 150 MG Oral Tablet |
| 1161290 | efavirenz Pill |
| 639888 | emtricitabine 200 MG / tenofovir disoproxil fumarate 300 MG Oral Tablet [Truvada] |
| 2374563 | dolutegravir 5 MG [Tivicay] |
| 1721615 | cobicistat 150 MG / elvitegravir 150 MG / emtricitabine 200 MG / tenofovir alafenamide 10 MG [Genvoya] |
| 597728 | ritonavir 50 MG |
| 1744005 | emtricitabine 133 MG / tenofovir disoproxil fumarate 200 MG Oral Tablet |
| 1551991 | cobicistat Pill |
| 406017 | emtricitabine Oral Capsule |
| 1741730 | emtricitabine / rilpivirine / tenofovir alafenamide Oral Product |
| 1600701 | cobicistat / darunavir Oral Product |
| 213460 | abacavir 300 MG Oral Tablet [Ziagen] |
| 2587899 | {20 (nirmatrelvir 150 MG Oral Tablet) / 10 (ritonavir 100 MG Oral Tablet) } Pack [Paxlovid 5-Day] |
| 670025 | darunavir 600 MG |
| 1999670 | bictegravir / emtricitabine / tenofovir alafenamide Oral Tablet [Biktarvy] |
| 1433876 | dolutegravir Oral Tablet [Tivicay] |
| 2587898 | {20 (nirmatrelvir 150 MG Oral Tablet) / 10 (ritonavir 100 MG Oral Tablet) } Pack |
| 1161885 | emtricitabine / tenofovir disoproxil Oral Product |
| 1546886 | abacavir / dolutegravir / lamivudine Oral Tablet |
| 1989502 | dolutegravir 50 MG / rilpivirine 25 MG [Juluca] |
| 1421195 | raltegravir Pill |
| 1236632 | darunavir 100 MG/ML Oral Suspension [Prezista] |
| 1741736 | emtricitabine / rilpivirine / tenofovir alafenamide Oral Tablet [Odefsey] |
| 402093 | atazanavir 200 MG Oral Capsule [Reyataz] |
| 573348 | abacavir 300 MG [Ziagen] |
| 824337 | darunavir 400 MG |
| 1102273 | rilpivirine 25 MG Oral Tablet |
| 1546887 | abacavir / dolutegravir / lamivudine |
| 744842 | raltegravir 400 MG Oral Tablet |
| 2055807 | doravirine / lamivudine / tenofovir disoproxil Oral Tablet |
| 1551990 | cobicistat Oral Product |
| 2598352 | abacavir 60 MG / dolutegravir 5 MG / lamivudine 30 MG Tablet for Oral Suspension [Triumeq] |
| 1421179 | raltegravir Oral Product |
| 1721610 | cobicistat / elvitegravir / emtricitabine / tenofovir alafenamide Pill |
| 1600707 | cobicistat / darunavir Oral Tablet [Prezcobix] |
| 2122523 | dolutegravir / lamivudine Oral Tablet [Dovato] |
| 2055808 | doravirine / lamivudine / tenofovir disoproxil |
| 2475412 | rilpivirine Injectable Product |
| 1235585 | raltegravir potassium |
| 1433871 | dolutegravir Pill |
| 1989496 | dolutegravir / rilpivirine Oral Product |
| 2043464 | efavirenz 600 MG / lamivudine 300 MG / tenofovir disoproxil fumarate 300 MG Oral Tablet [Symfi] |
| 214671 | lamivudine / zidovudine |
| 2043322 | 1.33 ML ibalizumab-uiyk 150 MG/ML Injection [Trogarzo] |
| 1102287 | rilpivirine hydrochloride |
| 242679 | abacavir 300 MG Oral Tablet |
| 616147 | emtricitabine Oral Solution [Emtriva] |
| 352230 | lamivudine 300 MG Oral Tablet [Epivir] |
| 2374564 | dolutegravir Tablet for Oral Suspension [Tivicay] |
| 1721616 | cobicistat / elvitegravir / emtricitabine / tenofovir alafenamide Oral Tablet [Genvoya] |
| 729582 | lamivudine / stavudine Oral Tablet |
| 1551992 | cobicistat Oral Tablet |
| 1744006 | emtricitabine 133 MG / tenofovir disoproxil fumarate 200 MG [Truvada] |
| 664742 | atazanavir 300 MG [Reyataz] |
| 664740 | atazanavir 300 MG |
| 1741735 | emtricitabine 200 MG / rilpivirine 25 MG / tenofovir alafenamide 25 MG [Odefsey] |
| 1161886 | emtricitabine / tenofovir disoproxil Pill |
| 1601660 | atazanavir 300 MG / cobicistat 150 MG Oral Tablet [Evotaz] |
| 1600702 | cobicistat / darunavir Pill |
| 2380548 | fostemsavir 600 MG |
| 1306294 | cobicistat 150 MG / elvitegravir 150 MG / emtricitabine 200 MG / tenofovir disoproxil fumarate 300 MG [Stribild] |
| 205292 | efavirenz 50 MG Oral Capsule |
| 374642 | lopinavir / ritonavir Oral Capsule |
| 2584351 | bictegravir 30 MG |
| 2055766 | doravirine 100 MG Oral Tablet [Pifeltro] |
| 565134 | ritonavir 100 MG [Norvir] |
| 1857917 | maraviroc 75 MG Oral Tablet [Selzentry] |
| 643069 | efavirenz / emtricitabine / tenofovir disoproxil Oral Tablet [Atripla] |
| 1989503 | dolutegravir / rilpivirine Oral Tablet [Juluca] |
| 1747694 | emtricitabine / tenofovir alafenamide Oral Tablet [Descovy] |
| 2055760 | doravirine 100 MG Oral Tablet |
| 1433870 | dolutegravir Oral Product |
